# Major depression disorder may causally associate with the increased breast cancer risk: Evidence from two‐sample mendelian randomization analyses

**DOI:** 10.1002/cam4.5043

**Published:** 2022-07-19

**Authors:** Qian Ren, Fangxiu Luo, Sheng Ge, Peizhan Chen

**Affiliations:** ^1^ Department of Clinical Nutrition Shanghai Jiao Tong University Affiliated Sixth People's Hospital Shanghai China; ^2^ Department of Pathology Ruijin Hospital, Shanghai Jiao Tong University School of Medicine Shanghai China; ^3^ Department of General Surgery Ruijin Hospital, Shanghai Jiao Tong University School of Medicine Shanghai China

**Keywords:** breast cancer, major depression disorder, mendelian randomization, single‐nucleotide polymorphism

## Abstract

**Introduction:**

Major depression disorder (MDD) has been associated with increased breast cancer risk in epidemiological studies; however, it is still unknown whether this association is causal or not. The aim of this study is to determine the causal relationship between MDD and breast cancer risk.

**Methods:**

Two‐sample Mendelian randomization (MR) analyses with 92 single‐nucleotide polymorphisms (SNPs) significantly associated with MDD as instrumental variables (IVs) were performed. Effects of these SNPs on breast cancer in women were estimated in the Breast Cancer Association Consortium (122,977 cases and 105,974 controls) using inverse variance weighted (IVW), weighted median and multivariable MR models. Heterogeneity and pleiotropy effects were assessed based on IVW and MR‐Egger regression model, respectively.

**Results:**

An 8.7% increased risk of overall breast cancer [odds ratio (*OR*) = 1.087; 95% confidence interval (*CI*) 1.011–1.170; *P* = 0.025] per log‐odds ratio increment of MDD risk based on the IVW model was noticed. Similar results were obtained with the multivariable MR model (*OR* = 1.118, 95% *CI* = 1.010–1.237; *P* = 0.031). An increment but not statistically significant causality association was noticed between MDD and risk of ER+ (*OR* = 1.098, 95% *CI* = 0.984–1.227; *P* = 0.093) or ER‐ (*OR* = 1.129, 95% *CI* = 0.982–1.297; *P* = 0.089) breast cancer under multivariable MR model. No significant pleiotropy effects were observed for the IVs in the two‐sample MR studies.

**Conclusions:**

The results suggested that a genetic predisposition of MDD is causally associated with overall breast cancer risk; however, the underlying biological mechanisms are worthy of further study.

## Graphical abstract

Using the two‐sample Medenlian Randomization analysis methods, we found a causal association between major depression disorder and breast cancer risk in women

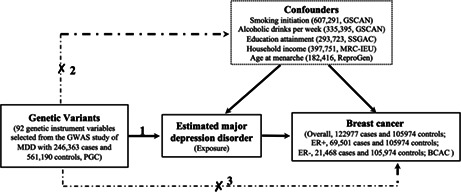

## 1 INTRODUCTION

Breast cancer is one of the most common cancer and also the leading cause of cancer deaths in women.^1^ It was estimated a total of 2.1 million new breast cancer cases and 0.63 million deaths caused by breast cancer globally in 2018.^2^ In a systemic analysis of the global burden of disease study, breast cancer played an important role in disability‐adjusted life years (DALYs) along with a rising trend in most countries.^3^ Previous studies have reported that the genetic factors, physical activities, nutritional factors, and exposure to specific environmental pollutions are associated with the increased risk of breast cancer development^4^; however, these analyses remain vulnerable to study design, and more studies are needed to identify the etiology factors for breast cancer.

Among the potential factors that may affect the risk of developing cancer, psychosocial factors have drawn an increasing attention.^5^ Depression is one of the most common psychosocial factors for patients with mood disorders, which was estimated to influence about 121 million people worldwide.^6^ A recent meta‐analysis has suggested that depression was associated with increased mortality of breast cancer^7^, ^8^, ^9^; however, the results were not always consistent.^10^ Because of the short following‐up time, incomplete or unreliable determination of depression status at baseline, and insufficient confounding control in the study design, the association between depression and breast cancer is still under debate.^11^ Therefore, more well‐designed studies are needed to elucidate the causal associations between the depression and breast cancer risk.

The Mendelian randomization (MR) analysis, which uses germline genetic variation as the instrumental variable (IVs) of the potential exposure to evaluate the associations between the modifiable exposure and the outcomes, can be applied to make a causal inference.^12^ Unlike traditional observational epidemiology, MR analysis could overcome the risk of bias caused by undetermined confoundings, reverse causality and measurement error.^13^ MR study has been recognized as analogous with the randomized controlled trail (RCT) studies in assessing the causal relationship between the exposures and the outcomes,^14^ but it requires less time and expense and may address the questions that RCT studies are unable to ask.^15^ A recent genome‐wide association meta‐analysis has been performed by Howard et al. who identified 102 independent single‐nucleotide polymorphisms (SNPs) that significantly associated with major depression disease (MDD), and they evaluated the causal associations between MDD and 41 types of diseases or diseases‐related traits using MR analysis methods^16^; however, whether MDD is causally associated with breast cancer risk has not yet determined. In the current study, we applied the genetic variations significantly associated with MDD as the genetic instrumental variables (IVs) to evaluate the causal relationship between MDD and breast cancer risk, which may provide novel intervention methods for breast cancer in future.

## 2 MATERIALS AND METHODS

The study was performed based on the summary‐level data of public genome‐wide association studies (GWASs), and it is not necessary to be approved by the ethic committee in our institute. Consents from the participants were assumed to be obtained by the individual GWAS.

### 2.1 Study design and data sources

**FIGURE 1:**
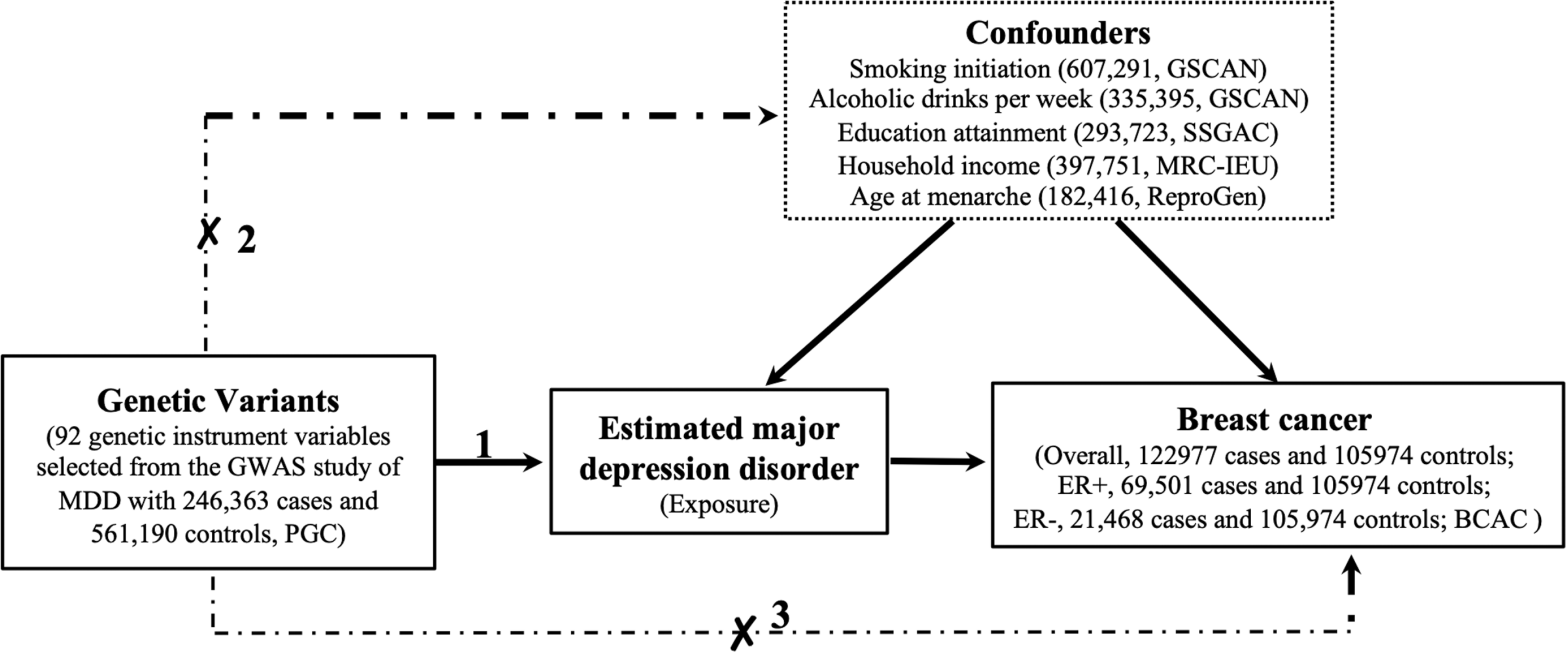
The study assumptions of the two‐sample Mendelian Randomization analysis between MDD and breast cancer. The assumptions including: (1) the genetic instrumental variables (IVs) should be significantly associated with MDD; (2) the genetic IVs should not be associated with other exposure‐results confounding factors; (3) the genetic IVs must only be through MDD and not through any other confounders to influence the breast cancer risk. The dotted lines indicate violate of the assumptions. BCAC, Breast Cancer Association Consortium; ER, Estrogen Receptor; GSCAN, GWAS and Sequencing Consortium of Alcohol and Nicotine use; MRC‐IEU, MRC Integrative Epidemiology Unit; PGC, Psychiatric Genomics Consortium; ReproGen, Reproductive Genetics Consortium; SSGAC, Social Science Genetic Association Consortium.

We applied the two‐sample MR study based on the public summary‐level data derived from the GWASs to evaluated the causal association between MDD and breast cancer risk. Genetic IVs were derived from the GWAS performed by Howard et al.,^16^ and their associations with overall breast cancer, ER+ or ER‐ risk were assessed using the summary‐level data from a GWAS study with 228,951 women including 122,977 breast cancer (69,501 ER+ and 21,468 ER‐ patients) cases and 105,974 controls of European ancestry from the Breast Cancer Association Consortium (BCAC).^17^ To assess the influences of the potential confounders, we also performed the multivariable MR (MVMR) studies to assess the causal association between MDD and breast cancer with the adjustment for smoking initiation, alcoholic drinks per week, education (years of schooling), average household income (before tax), and age at menarche. Summary statistics data for smoking intiation and alcoholic drinks per week were extracted from meta‐analysis of GWASs conducted by the GWAS and Sequencing Consortium of Alcohol and Nicotine use (GSCAN) consortium.^18^ Summary level data of education attainment represented as the number of years of schooling were derived from a GWAS performed by Social Science Genetic Association Consortium (SSGAC).^19^ SNPs significantly associated with the average household income before tax were extracted from the MRC‐IEU UK Biobank GWAS of European ancestry with 397,751 response individuals.^20^, ^21^ Summary level data related to the associations between SNPs and age at menarche are extracted from the GWAS performed by ReproGen Consortium.^22^ Detailed information for the included GWASs was provided in Table S1, and the overall study design was shown in Figure 1.

### 2.2 Instrumental variables selection and validation

According to the MR study design, the IVs used to assess causal relationship between MDD and breast cancer risk should meet the following assumptions: (1) the IVs should be significantly associated with MDD risk; (2) the IVs should not be associated with other potential confounding factors; (3) there is no direct association between the IVs and breast cancer, or through any other way except the exposure (MDD) to correlate with breast cancer (Figure 1). Here, single‐nucleotide polymorphisms (SNPs) from a large genome‐wide meta‐analysis performed by Psychiatric Genomics Consortium (PGC) consortium were recognized as the potential genetic IVs.^16^ As reported by Howard et al.,^16^ 102 independent variants locate on 101 genomic loci that were associated with depression were identified in the meta‐analysis with a total of 807,553 individuals including 246,363 cases and 561,190 controls that derived from three GWAS of depression in European ancestry.^16^ All these SNPs have reached the significant genome‐wide significance (*P* < 5 × 10^−8^), and the base pair positions of them were identified by clumping all associated variants across a 3 Mb window on chromosome (linkage disequilibrium *r* ^*2*^ < 0.1) and have merged any overlapping clumps, indicating these IVs met the assumptions 1 and 2 of the two‐sample MR study design. Of them, four SNPs including rs78337797, rs56314503, rs10774600, and rs3213572 were not identified in the BCAC GWAS summary‐level data for breast cancer risk or with high LD proxy SNPs (*r* ^*2*^ > 0.80). Another six SNPs including rs12052908, rs1933802, rs2029865, rs2247523, rs263645, and rs2876520 were discarded for being palindromic with intermediate allele frequencies (Table 1). Thus, a total of 92 validated IVs was applied in the current two‐sample MR study (Table 1). To calculate *R* ^*2*^, we used the following formula: (2 × EAF [1‐EAF] × β^2^), where EAF is the effect allele frequency, β is the estimated genetic effect on MDD. We calculated the *F*‐statistic to assess the strength of each IVs using the formulation: F = (*R* ^*2*^ × [n − 1 ‐ k])/([1 ‐ *R* ^*2*^] × k) as reported,^23^ where *R* ^*2*^ was calculated as proportion of variance in the phenotype explained by genetic variants, k is the number of IVs, and n is the sample size. An *F*‐statistic >10 has been recognized as a robust IV and recommended to be used in MR study.^23^ The *F*‐statistic value of these genetic IVs ranged from 147 to 532 indicating sufficient strength of the IVs in the two‐sample MR studies (Table 1).

**TABLE 1:** Summary information on MDD‐associated SNPs used as genetic instrumental variables (IVs) for the two‐sample Mendelian randomization (MR) analyses

SNP	Chr	Position	Effect allele	Other allele	EAF	β	SE	*P*‐value	*R* ^*2*^	*F*‐statistic
rs301799	1	8,489,302	T	C	0.5694	−0.0250	0.0035	1.36 × 10^−12^	1.08 × 10^−3^	247.571
rs1002656	1	37,192,741	T	C	0.7033	−0.0266	0.0038	3.74 × 10^−12^	1.04 × 10^−3^	238.531
rs1466887	1	37,709,328	T	C	0.5511	−0.0199	0.0036	4.12 × 10^−8^	6.91 × 10^−4^	158.258
rs11579246	1	50,559,162	A	G	0.9067	0.0381	0.0061	5.71 × 10^−10^	8.66 × 10^−4^	198.380
rs1890946	1	52,342,427	T	C	0.4671	−0.0235	0.0035	2.68 × 10^−11^	9.69 × 10^−4^	222.078
rs10789214	1	67,146,817	T	C	0.5661	0.0193	0.0035	4.44 × 10^−8^	6.45 × 10^−4^	147.799
rs2568958	1	72,765,116	A	G	0.6156	0.0373	0.0036	8.47 × 10^−25^	2.32 × 10^−3^	532.085
rs10890020	1	73,668,836	A	G	0.5156	−0.0277	0.0035	4.03 × 10^−12^	1.35 × 10^−3^	309.627
rs113188507	1	80,809,636	A	G	0.2838	0.0221	0.0039	1.87 × 10^−8^	7.00 × 10^−4^	160.366
rs10913112	1	175,913,828	T	C	0.3767	−0.0264	0.0036	3.40 × 10^−13^	1.15 × 10^−3^	264.386
rs72710803	1	177,428,018	A	C	0.9121	−0.0410	0.0062	5.29 × 10^−11^	9.50 × 10^−4^	217.726
rs169235	1	181,740,924	A	G	0.753	−0.0229	0.0041	2.98 × 10^−8^	6.88 × 10^−4^	157.559
rs17641524	1	197,704,717	T	C	0.2091	−0.0320	0.0043	1.52 × 10^−13^	1.19 × 10^−3^	273.601
rs12052908	2	22,503,044	A	T	0.5325	−0.0220	0.0035	4.44 × 10^−10^	8.49 × 10^−4^	194.646
rs1568452	2	58,012,833	T	C	0.3851	0.0248	0.0036	8.12 × 10^−12^	1.03 × 10^−3^	235.290
rs7585722	2	86,819,128	T	C	0.8458	−0.0269	0.0048	2.68 × 10^−8^	6.65 × 10^−4^	152.452
rs1226412	2	157,111,313	T	C	0.7917	0.0256	0.0043	3.46 × 10^−9^	7.62 × 10^−4^	174.590
rs62188629	2	208,044,470	A	G	0.3136	0.0236	0.0038	7.13 × 10^−10^	8.45 × 10^−4^	193.676
rs4346585	3	44,736,493	T	C	0.696	−0.0236	0.0038	7.13 × 10^−10^	8.31 × 10^−4^	190.373
rs13084037	3	49,214,066	A	G	0.774	−0.0245	0.0042	7.08 × 10^−9^	7.40 × 10^−4^	169.616
rs7624336	3	53,244,151	T	G	0.2087	0.0238	0.0043	3.96 × 10^−8^	6.60 × 10^−4^	151.110
rs141954845	3	61,192,911	A	G	0.388	0.0229	0.0037	8.15 × 10^−10^	8.78 × 10^−4^	201.167
rs6783233	3	117,509,984	T	C	0.2833	0.0218	0.0039	2.90 × 10^−8^	6.80 × 10^−4^	155.875
rs1095626	3	157,977,962	T	C	0.5799	−0.0264	0.0035	7.13 × 10^−14^	1.20 × 10^−3^	274.319
rs7685686	4	3,207,142	A	G	0.5753	0.0202	0.0036	2.57 × 10^−8^	7.03 × 10^−4^	161.050
rs34937911	4	42,110,353	T	C	0.8838	0.0304	0.0055	4.13 × 10^−8^	6.69 × 10^−4^	153.315
rs45510091	4	123,186,393	A	G	0.9472	0.0448	0.0080	1.83 × 10^−8^	7.08 × 10^−4^	162.149
rs35553410	4	131,237,381	T	C	0.7462	−0.0244	0.0040	1.42 × 10^−9^	7.95 × 10^−4^	182.146
rs7659414	4	177,350,956	A	C	0.5782	−0.0201	0.0035	1.20 × 10^−8^	6.95 × 10^−4^	159.169
rs60157091	5	61,509,655	T	C	0.515	0.0200	0.0035	1.42 × 10^−8^	7.04 × 10^−4^	161.395
rs3099439	5	87,545,318	T	C	0.5288	−0.0276	0.0035	5.05 × 10^−15^	1.34 × 10^−3^	306.673
rs10061069	5	93,071,630	C	G	0.2212	−0.0275	0.0042	8.15 × 10^−11^	9.18 × 10^−4^	210.467
rs30266	5	103,972,357	A	G	0.3296	0.0308	0.0037	1.45 × 10^−16^	1.48 × 10^−3^	338.688
rs11135349	5	164,523,472	A	C	0.4713	−0.0295	0.0035	6.04 × 10^−17^	1.53 × 10^−3^	350.376
rs200949	6	27,835,435	A	G	0.8744	0.0480	0.0053	2.53 × 10^−19^	1.78 × 10^−3^	408.881
rs9363467	6	66,565,703	T	C	0.6035	0.0237	0.0036	6.44 × 10^−11^	9.48 × 10^−4^	217.135
rs7758630	6	101,387,304	A	T	0.4051	−0.0225	0.0036	5.56 × 10^−10^	8.60 × 10^−4^	197.094
rs1933802	6	105,365,891	C	G	0.4536	−0.0223	0.0035	2.57 × 10^−10^	8.69 × 10^−4^	199.111
rs2876520	6	142,996,618	C	G	0.5271	−0.0230	0.0036	2.29 × 10^−10^	9.30 × 10^−4^	213.024
rs725616	6	147,950,422	T	C	0.3644	0.0204	0.0036	1.87 × 10^−8^	6.80 × 10^−4^	155.705
rs2029865	6	165,121,844	A	T	0.4534	−0.0201	0.0035	1.20 × 10^−8^	7.06 × 10^−4^	161.743
rs3823624	7	2,110,346	T	C	0.8067	0.0272	0.0045	1.99 × 10^−9^	8.13 × 10^−4^	186.370
rs2043539	7	12,253,880	A	G	0.4177	0.0273	0.0035	9.89 × 10^−15^	1.28 × 10^−3^	292.880
rs2247523	7	82,454,404	C	G	0.5319	−0.0207	0.0035	4.38 × 10^−9^	7.52 × 10^−4^	172.344
rs16887442	7	82,936,909	T	C	0.4347	0.0203	0.0035	8.62 × 10^−9^	7.14 × 10^−4^	163.585
rs58104186	7	109,099,919	A	G	0.4689	0.0237	0.0035	1.82 × 10^−11^	9.86 × 10^−4^	225.980
rs7807677	7	117,502,574	T	C	0.5505	0.0237	0.0035	1.82 × 10^−11^	9.80 × 10^−4^	224.543
rs7837935	8	65,562,019	T	G	0.1522	−0.0292	0.0049	3.34 × 10^−9^	7.76 × 10^−4^	177.732
rs67436663	8	71,347,626	C	G	0.2402	−0.0259	0.0042	9.37 × 10^−10^	8.63 × 10^−4^	197.776
rs1354115	9	2,983,774	A	C	0.6243	0.0210	0.0036	7.08 × 10^−9^	7.29 × 10^−4^	167.093
rs1982277	9	11,513,019	T	C	0.7594	0.0279	0.0041	1.45 × 10^−11^	1.00 × 10^−3^	229.770
rs263645	9	17,016,503	A	T	0.5438	0.0221	0.0035	3.70 × 10^−10^	8.54 × 10^−4^	195.740
rs3793577	9	23,737,627	A	G	0.4665	−0.0229	0.0035	8.41 × 10^−11^	9.20 × 10^−4^	210.846
rs59283172	9	25,232,978	A	G	0.1069	−0.0329	0.0057	1.02 × 10^−8^	7.29 × 10^−4^	166.938
rs34653192	9	31,124,452	C	G	0.3196	−0.0229	0.0038	2.23 × 10^−9^	8.04 × 10^−4^	184.220
rs7030813	9	36,999,369	T	C	0.3736	0.0253	0.0036	3.07 × 10^−12^	1.06 × 10^−3^	242.005
rs10817969	9	119,731,045	T	G	0.7173	0.0261	0.0039	3.11 × 10^−11^	9.74 × 10^−4^	223.163
rs913930	9	120,484,009	A	G	0.6433	−0.0208	0.0037	2.42 × 10^−8^	7.00 × 10^−4^	160.371
rs2670139	9	126,634,255	T	C	0.7609	−0.0266	0.0041	1.21 × 10^−10^	9.07 × 10^−4^	207.959
rs997934	10	1,795,194	T	C	0.3795	0.0198	0.0036	4.81 × 10^−8^	6.51 × 10^−4^	149.128
rs1021363	10	106,610,839	A	G	0.3547	0.0303	0.0037	4.41 × 10^−16^	1.48 × 10^−3^	339.536
rs1448938	11	30,892,824	A	G	0.4171	0.0214	0.0035	1.30 × 10^−9^	7.85 × 10^−4^	179.868
rs2509805	11	57,650,796	T	C	0.3209	0.0220	0.0038	9.17 × 10^−9^	7.44 × 10^−4^	170.387
rs198457	11	61,471,678	T	C	0.1925	−0.0292	0.0046	2.99 × 10^−10^	9.34 × 10^−4^	214.116
rs58621819	11	65,314,830	A	T	0.7903	−0.0245	0.0043	1.57 × 10^−8^	7.01 × 10^−4^	160.696
rs7117514	11	70,544,937	A	G	0.5417	−0.0204	0.0035	7.29 × 10^−9^	7.28 × 10^−4^	166.899
rs7932640	11	88,744,425	T	C	0.4417	0.0281	0.0035	1.62 × 10^−15^	1.37 × 10^−3^	314.610
rs61902811	11	113,370,758	A	G	0.3682	−0.0257	0.0036	1.40 × 10^−12^	1.08 × 10^−3^	248.232
rs2187490	11	118,713,180	T	G	0.9106	−0.0338	0.0061	3.82 × 10^−8^	6.56 × 10^−4^	150.236
rs57344483	11	127,022,560	A	G	0.9259	−0.0380	0.0068	1.82 × 10^−8^	6.99 × 10^−4^	160.041
rs78337797	12	23,987,925	T	G	0.8781	0.0306	0.0055	3.37 × 10^−8^	7.07 × 10^−4^	161.910
rs56314503	12	84,465,022	T	G	0.7487	−0.0254	0.0040	2.95 × 10^−10^	8.56 × 10^−4^	196.096
rs10774600	12	110,741,356	T	C	0.1656	−0.0267	0.0048	3.39 × 10^−8^	6.95 × 10^−4^	159.125
rs3213572	12	121,205,078	A	G	0.4745	0.0217	0.0035	7.61 × 10^−10^	8.28 × 10^−4^	189.682
rs1409379	13	31,907,741	T	C	0.7641	0.0249	0.0041	1.67 × 10^−9^	7.88 × 10^−4^	180.538
rs1343605	13	53,647,048	A	C	0.384	0.0313	0.0036	6.23 × 10^−18^	1.63 × 10^−3^	374.453
rs9592461	13	66,941,792	A	G	0.4874	0.0216	0.0035	9.10 × 10^−10^	8.22 × 10^−4^	188.308
rs9545360	13	80,826,373	A	C	0.1807	−0.0271	0.0046	5.02 × 10^−9^	7.67 × 10^−4^	175.642
rs4772087	13	99,115,041	T	C	0.3732	0.0227	0.0036	3.91 × 10^−10^	8.50 × 10^−4^	194.725
rs61990288	14	42,074,726	A	G	0.5083	−0.0260	0.0035	1.68 × 10^−13^	1.19 × 10^−3^	272.966
rs1956373	14	60,141,822	T	G	0.7436	−0.0226	0.0040	2.06 × 10^−8^	6.87 × 10^−4^	157.309
rs1152578	14	64,697,037	T	C	0.4357	−0.0218	0.0035	6.36 × 10^−10^	8.24 × 10^−4^	188.759
rs1045430	14	75,130,235	T	G	0.4792	−0.0253	0.0035	7.31 × 10^−13^	1.13 × 10^−3^	258.085
rs10149470	14	104,017,953	A	G	0.4869	−0.0267	0.0035	3.72 × 10^−14^	1.26 × 10^−3^	287.749
rs8037355	15	37,643,831	T	C	0.5556	−0.0233	0.0035	3.94 × 10^−11^	9.45 × 10^−4^	216.551
rs34488670	15	47,684,936	T	C	0.7887	−0.0252	0.0043	6.03 × 10^−9^	7.46 × 10^−4^	170.962
rs7193263	16	6,315,880	A	G	0.6679	−0.0239	0.0038	4.33 × 10^−10^	8.93 × 10^−4^	204.683
rs7198928	16	7,666,402	T	C	0.6159	0.0239	0.0036	4.45 × 10^−11^	9.53 × 10^−4^	218.305
rs7200826	16	13,066,833	T	C	0.2551	0.0280	0.0040	3.74 × 10^−12^	1.05 × 10^−3^	240.685
rs56887639	16	13,755,530	A	G	0.7264	−0.0278	0.0039	1.51 × 10^−12^	1.08 × 10^−3^	248.148
rs12923444	16	21,639,710	A	C	0.5625	−0.0214	0.0035	1.30 × 10^−9^	7.95 × 10^−4^	182.063
rs75581564	17	27,363,750	A	G	0.1165	0.0301	0.0054	3.17 × 10^−8^	6.58 × 10^−4^	150.640
rs12967855	18	35,138,245	A	G	0.3295	0.0265	0.0037	1.18 × 10^−12^	1.09 × 10^−3^	250.655
rs7227069	18	50,731,802	A	G	0.4326	0.0238	0.0035	1.50 × 10^−11^	9.80 × 10^−4^	224.619
rs62091461	18	52,488,672	T	C	0.2274	−0.0254	0.0042	1.95 × 10^−9^	7.99 × 10^−4^	183.108
rs12966052	18	52,751,639	C	G	0.1805	−0.0314	0.0046	1.25 × 10^−11^	1.03 × 10^−3^	235.617
rs12967143	18	53,099,012	C	G	0.6984	−0.0312	0.0038	3.70 × 10^−16^	1.44 × 10^−3^	331.297
rs7241572	18	77,580,712	A	G	0.201	0.0280	0.0044	2.70 × 10^−10^	8.88 × 10^−4^	203.406
rs33431	19	30,939,989	T	C	0.6144	0.0198	0.0036	4.81 × 10^−8^	6.55 × 10^−4^	150.036
rs143186028	20	39,997,404	T	G	0.1778	0.0277	0.0046	2.29 × 10^−9^	7.91 × 10^−4^	181.201
rs12624433	20	44,680,853	A	G	0.2584	0.0233	0.0040	7.44 × 10^−9^	7.34 × 10^−4^	168.058
rs5995992	22	41,487,218	T	C	0.7155	−0.0266	0.0039	1.30 × 10^−11^	1.02 × 10^−3^	232.689

Abbreviations: EAF, effect allele frequency; SE, standard error; SNP, single‐nucleotide polymorphism.

### 2.3 Genetic associations between IVs and breast cancer risk

The summary‐level data for the associations between each genetic IVs with total, ER+ and ER‐ breast cancer patients were retrieved through the MRC Integrative Epidemiology Unit (IEU) GWAS database (https://gwas.mrcieu.ac.uk/) using the TwoSampleMR package (version 0.5.6) of R (www.r‐project.org).^24^ Summary data for the associations between MDD associated genetic variants with breast cancer were obtained from a GWAS with 228,951 women (including 122,977 breast cancer cases and 105,974 controls) of European ancestry performed by the Breast Cancer Association Consortium (BCAC) with study ID “ieu‐a‐1126”.^17^ Of the patients, 69,501 were diagnosed as ER+ breast cancer and 21,468 as ER‐ breast cancer patients, and summary‐level data were extracted with the study ID “ieu‐a‐1127” and “ieu‐a‐1128”, respectively. When SNP for the exposed phenotype is missing in summary statistics of breast cancer risk, it was replaced with another proxy SNPs that is in high linkage disequilibrium (*r* ^*2*^ > 0.80) with validated IVs as determined using the 1000 genomes reference panel in European ancestry individuls. None of the IVs is significantly associated with breast cancer at the genome‐wide significance (*P* < 5 × 10^−8^; Table S2), indicating that these IVs met the assumption 3 of two‐sample MR study design.

### 2.4 Genetic instrumental variables for confounders

To account for the confounders including smoking, alcohol, age at menarche, education attainment, and incoming in MVMR study, we extracted SNPs that significantly associated (*P* < 5 × 10^−8^) with smoking initiation (*n* = 607,291, GSCAN),^18^ alcoholic drinks per week (*n* = 335,394, GSCAN),^18^ years of schooling (n = 293,723, SSGAC),^19^ average household income before tax (*n* = 397,751, MRC‐IEU UK Biobank),^20^, ^21^ and age at menarche (*n* = 182,416, ReproGen) through querying the the IEU GWAS database (https://gwas.mrcieu.ac.uk) using the TwoSampleMR package.^24^ We further pruned the results to exclude all SNPs with a pairwise linkage disequirium (LD) *r* ^2^ > 0.001 in a 10,000 kb window of genomic region as final IVs.

### 2.5 Statistical power calculation

The pos hoc statistical power was calculated using an online tool at http://cnsgenomics.com/shiny/mRnd/.^25^ The 92 SNP MDD instruments explained an estimated 2.448% of phenotypic variability. Given a type 1 error of 5%, we had sufficient power (> 80%) when the expected *OR* was 1.090 for overall breast cancer (122,977 cases and 105,974 controls) per log‐odds ratio increase with MDD in women. In the stratification analysis, we noticed the statistical power was 0.46 and 0.55 when the expected *OR* was 1.059 and 1.101 for ER+ (69,501 cases and 105,974 controls) and ER‐ (21,468 cases and 105,974 controls) breast cancer per log‐odds ratio increase with MDD in women, respectively. Statistical power estimates for the 92 genome‐wide significant SNP instruments by breast cancer subtypes are presented in Table S3.

### 2.6 Statistical analyses

In order to determine the causal relationship between MDD and breast cancer risk, we used the conventional inverse variance weighted (IVW) method, fixed‐effects IVW model, as well as the weighted median method to generate the main MR estimates. To account for the potential confounders in assessing MDD and breast cancer risk by the genetic IVs, we also performed the MVMR analysis adjusting for smoking, alcohol intake, education atttainment, income, and age at menarche. The genome‐wide significant signals of these exposures assessed independent of MDD‐related variants (*R* ^*2*^ LD <0.1 based on the 1000 Genome panel reference) were included in the models (Table S4).^26^ Due to the missing of 23andMe data, assocaitions between the IVs of confounders and MDD were assessed in meta‐analysis of UKBiobank and PGC with 170,756 cases and 329,443 cases.^16^ We applied the MR‐Egger method to determine the evidence of pleiotropic effects for these genetic IVs, which occurs when the variants has an effect on disease outside of its effect on the exposure (MDD) in MR studies.^27^ Intercepts that significantly deviate from the origin indicating an evidence of potential pleiotropy effects. We also performed the MR pleiotropy residual sum and outlier (MR‐PRESSO) test to identify any outlier variants that challenge the pleiotropy effects assumption (*P* < 0.05), and the effect estimates were recalculated after remove these outliers.^28^ Heterogeneity between the causal estimates inferred by the IVs was assessed using Cochran's Q statistic under the conventional IVW method. The leave‐one‐out sensitivity method was performed to evaluate whether the causality estimate was affected by a single SNP under conventional IVW model.^29^ We also performed MR‐Steiger directionality test to determine whether the assumption that exposure (MDD) causes outcome (breast cancer risk) is valid.^30^ The TwoSampleMR package (version 0.5.6) implemented in R (version 3.6.3) was used to obtain the data and perform all the two‐sample MR analyses. All statistical tests were two‐sided and considered statistical significance when *P* < 0.05.^24^, ^31^ For multiple testings, the Bonferroni‐corrected *P* values below 0.017 (where α = 0.05/3 breast cancer outcomes) were considered strong evidence of associations and *P* values between 0.017 and 0.05 as suggestive evidence of associations.

## 3 RESULTS

### 3.1 Causal relationship between MDD and risks of breast cancer

**FIGURE 2:**
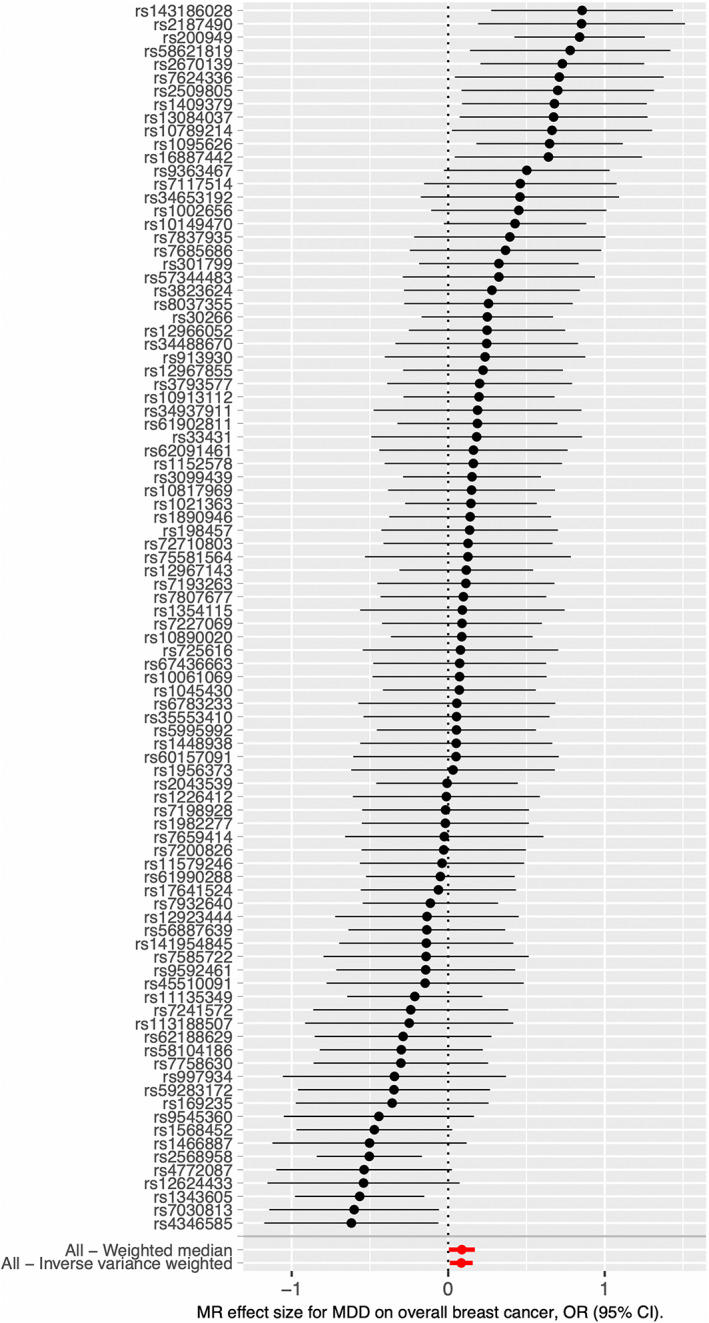
Forest plot of the two‐sample MR analysis results between MDD and the risk of breast cancer using conventional inverse variance weighted (IVW) and weighted median methods. Each black dot and horizontal line represented the causal effect obtained by Wald ratio method and 95% *CI* for individual IV, respectively. The red dots and horizontal lines at the bottom represent the overall causal effects obtained by different methods and 95% *CI*, respectively. X‐axis is shown in natural logarithmic scale.

Based on the IVW model of two‐sample MR studies, we found that women were associated with an 8.7% increased risk of overall breast cancer (*OR* = 1.087, 95% *CI* = 1.011–1.170; *P* = 0.025; Table 2) per log‐odds ratio increment of MDD. The causal estimate inferred by individual IVs was shown in funnel plot (Figure 2) as well as the scatterplot (Figure S1). Significant heterogeneity was noticed for the causal estimates between IVs based on the IVW model (*P*‐heterogeneity <0.001; Table 2). Similarly, the causal relationship between MDD and breast cancer was also established under weighted median model (*OR* = 1.091, 95% *CI* = 1.002–1.189; *P* = 0.045; Table 2 and Figure 2) as suggested by the MR regression slopes in scatterplot (Figure S1). However, the association did not reach the statistical significance (*P* < 0.017) for multiple testings. The leave‐one‐out analyses identified no individual IV largely affect the causal magnitude between MDD and the risks of total breast cancer under the IVW model (Figure S2). The MR‐Steiger directionality test suggested the causal direction by MDD on overall breast cancer risk is valid (*P* < 0.001).

**TABLE 2:** Estimates of the two‐sample MR analyses for causal associations between MDD and overall breast cancer risk

Outcomes	SNPs	Participants N (case/control)	MR estimates, per log‐odds ratio increment of MDD							Pleiotropy effects	
			IVW			Weighted median		MR‐PRESSO corrected		MR‐Egger regression Intercept	
			*OR* (95% *CI*)	*P*‐value	*P*‐_heter_	*OR* (95% *CI*)	*P*‐value	*OR* (95% *CI*)	*P*‐value	Intercept (SE)	*P*‐value
Breast cancer	92	228,951 (122,977/105,974)	1.087 (1.011–1.170)	0.025	< 0.001	1.091 (1.002–1.189)	0.046	1.090 (1.017–1.168)	0.016	−0.0004 (0.006)	0.946
ER+ Breast cancer	92	175,475 (69,501/105,974)	1.059 (0.969–1.157)	0.208	< 0.001	1.049 (0.945–1.165)	0.367	1.059 (0.974–1.152)	0.179	−0.0036 (0.0069)	0.599
ER‐ Breast cancer	92	127,442 (21,468/105,974)	1.163 (0.994–1.361)	0.060	0.303	1.100 (0.989–1.224)	0.078	1.101 (0.990–1.224)	0.081	0.000 (0.008)	0.991

Abbreviations: 95% *CI*, 95% confidential interval; ER, estrogen receptor; IVW, inverse variance weighted; MR, Mendelian Randomization; MR‐PRESSO, MR‐pleiotropy residual sum and outlier; *OR*, odds ratio; *P*‐heter, *P*‐heterogneity; SE, standard error; SNP, single‐nucleotide polymorphism.

According to MR‐Egger test, there was no significant pleiotropy effects of the genetic IVs (*P* = 0.946). When we applied the MR‐PRESSO analysis, a stronger association was observed between the MDD and breast cancer risk after excluding potential outlying pleiotropic IVs (*OR* = 1.090, 95% *CI* = 1.017–1.168), which reached the multiple testing significance threshold (*P* = 0.016). Further, the results from the MV analyses adjusting for potential confounders including regulatory smoking, alcoholic drinks per week, education attainment, household income (before tax), and age at menarche also suggested that MDD is associated with increased breast cancer (*OR* = 1.118, 95% *CI* = 1.010–1.237; *P* = 0.031).

### 3.2 Subtype stratification analysis the causal effects of MDD on ER+ or ER‐ breast cancer

To further explore the causal relationships between MDD and the risks of breast cancer of different pathological subtype, we performed the two‐sample MR studies based on the summary level data form the GWASs performed in ER+ and ER‐ breast cancer patients. No statistically significant association was observed between MDD and ER+ breast cancer as suggested by the IVW methods (*OR* = 1.059, 95% *CI* = 0.969–1.157; *P* = 0.208), weighted median methods and MR‐PRESSO corrected testing (Table 2). The causal estimate inferred by individual IVs was shown in scatterplot (Figure S3) as well as the funnel plot (Figure S4). There was a significant heterogeneity for the causal estimates between IVs for ER+ breast cancer based on the conventional IVW model (*P*‐heterogeneity <0.001) and no significant pleiotropy effects of the IVs in assessing causality association between MDD and ER+ breast cancer risk were noticed. The leave‐one‐out analyses identified no individual IV largely affect the causal relationship between MDD and ER+ breast cancer under the IVW model (Figure S5). Multivariable MR analysis adjusting for potential confounders found a marginal causality relationship between MDD and ER+ breast cancer (*OR* = 1.098, 95% *CI* = 0.984–1.227; *P* = 0.093).

For ER‐ breast cancer, we noticed a statistically marginal causality relationship with MDD as suggested by IVW (*OR* = 1.163, 95% *CI* = 0.994–1.361; *P* = 0.060), weighted median (*OR* = 1.100, 95% *CI* = 0.989–1.224; *P* = 0.078), and MR‐PRESSO analyses (*OR* = 1.101, 95% *CI* = 0.990–1.224; *P* = 0.081; Table 2). Causal estimates inferred by individual IVs was shown in scatterplot (Figure S6) as well as the funnel plot (Figure S7). No significant heterogeneity was noticed between the IVs under the IVW model (*P*‐heterogeneity = 0.303) and no evidence for the directional horizontal pleiotropy was identified in the MR‐Egger regression analysis (Table 2). We performed leave‐one‐out sensitivity analysis based on the conventional IVW model, and no individual IV largely affect the causal estimates between MDD and the risk of ER‐ breast cancer was noticed (Figure S8). The multivariable MR also suggesting a statistically marginal causality between MDD and ER‐ breast cancer (*OR* = 1.129, 95% *CI* = 0.982–1.297; *P* = 0.089).

## 4 DISCUSSION

By using the summarized statistical data from public GWASs, we performed a two‐sample MR study to evaluate the causal associations between MDD and breast cancer risk. We found a statistical causal association between a genetic predisposition of MDD and the risk of overall breast cancer. As breast cancer with distinct pathological status may caused by different etiology factors and show varied responses to clinical treatments,^32^ we performed stratification analysis between MDD and ER+ or ER‐ breast cancer; however, no statistically significant casuality was noticed due to relatively lower statistical power (< 80%) in stratification studies. Whether MDD has different influences on ER+ or ER‐ breast cancer also needs to be elucidated with more studies.

Previous observational studies have suggested that MDD might be associated with increased breast cancer risk^11^, ^33^, ^34^; however, the results were not always consistent.^10^ Discrepancy results may be due to the different diagnostic criteria of depression, the different sample size of people observed in the cohort, the different following‐up time,^35^, ^36^ and other confounding factors.^11^, ^37^ As most cancers have a longer incubation period, it is difficult for traditional epidemiology to establish the causal relationship between MDD and breast cancer risk. The loss of following‐up may vary depending on the state of cancer or depression, which may lead to biased estimates of association and standard error. Wrong misspecification in risk models may be another limitation, as other important confounding factors such as genetic susceptibility or congenital, and unmeasured psychological characteristics that have not been explained. Measurement methods of depression may influent the assessment of the exposure factors. The most commonly used Diagnostic Interview Schedule (DIS) test for severe depression has a relatively high specificity (more than 95% of test negatives are true negatives), but the estimated sensitivity is often difficult to exceed 40% (about 30% to 40%).^38^, ^39^ Therefore, misclassification of depression may contribute to the null association results in specific studies. Some studies often use the self‐reported cancer status of study participants as the outcome inclusion criterion. This measurement error will also weaken the estimation of association between MDD and breast cancer risk.^11^ At least but not the last, due to high median age at onset of breast cancer, related research is trapped in personnel, funding, and other factors often make it difficult to track the entire cohort or until the onset of cancer, which may distort the associations between MDD and breast cancer risk.^40^ Unlike these traditional epidemiological studies, we applied the two‐sample MR, which used genetic variants do not influence the outcomes via a different biological pathway from the exposure of interest, to control confounders. We conducted multiple sensitivity analyses to test for the influences of pleiotropy on our causal estimates, and robust results were observed according to these various tests. Moreover, design of MR analysis along with the MR Steiger test of directionality suggest a low probability of reverse causality in assessing the influences of MDD on breast cancer risk.^30^

Our two‐sample MR study supports the hypothesis that depression can lead to higher risk of breast cancer.^11^ Multiple biological mechanisms are hypothesized to mediate the potential beneficial role of MDD on breast cancer development. MDD is associated with higher circulating levels of inflammatory cytokines, such as interleukin (IL)‐6, IL‐8, IL‐1β, TNF‐α, soluble IL‐2 receptor (sIL‐2R), and C‐reactive protein (CRP), which promote cellular proliferation in breast tissue and have also been linked to development of breast cancer.^41^, ^42^ Continuous chronic inflammation status may underlie the cancer development caused by MDD.^43^ MDD have also been associated with a dysfunctional activation of the hypothalamic–pituitary adrenal (HPA) axis, which are risk factors for breast cancer development.^44^ As a stress hormone, cortisol is released from the adrenal glands when the adrenocorticotropic hormone in the blood is elevated. When cortisol is under continuous chronic stress and abnormal activation of the HPA axis, its metabolic level may be dysregulated. Therefore, the normal cortisol response pattern is destroyed under a long‐term stressor and the ability to respond to stress is destroyed.^45^ Cortisol also plays important roles in the activation of cell growth and cell cycle regulation signaling.^46^, ^47^, ^48^ Related studies indicated that flattening cortisol levels throughout the day is significantly associated with an increased risk of breast cancer.^49^, ^50^ Moreover, MDD has also been associated with a decreased immunosurveillance with increased risk of breast cancer. The depression augments the production of autoantibodies that against 16α‐OHE1‐ER through the generation of inflammatory conditions.^51^ Finally, emerging evidence suggests that the gut microbiome may play an important role in the MDD and breast cancer relationship. MDD patients have less *Faecalibacterium*, which inversely correlated with the severity of the depression,^52^ and the dysbiosis of the gut microbiome has been associated with increased risks of breast and cancer.^53^

An important assumption of MR is that the genetic IVs should not influence the outcome through a different biological pathway from the exposure of tested. Previous epidemiology studies suggested that MDD may lead to higher rates of smoking and alcohol intake.^54^, ^55^ Meta‐analysis of the observational epidemiological studies had suggested that smoking may weakly increase the breast cancer risk.^56^ Dimou et al. have observed a genetic predisposition to higher lifetime amount of smoking was positively associated with overall breast cancer (*OR* = 1.13, 95% *CI* = 1.00–1.26; *P* = 0.04). MR study performed by Howard et al.^16^ reported that MDD is causally associated with breast cancer risk factors including smoking and age at menarche. Although the MR study did not identify an association between genetically predicted alcohol consumption with breast cancer risk, the prospective cohort studies have suggested that higher alcohol intake was associated increased breast cancer risk.^57^ Moreover, socio‐economic status (SES) such as education level and household income were reported to be negatively associated with depressive symptoms,^58^, ^59^ and SES is associated with breast cancer susceptibility and prognosis of breast cancer patients in clinic.^60^ To account for such potential confounders that may mediate the causality between MDD and breast cancer, we performed MVMR to evaluate the causality relationship between MDD and breast cancer risk with the adjustments for regulatory smoking, alcohol intake, years of schooling, household income, and age at menarche. The MVMR results also observed an increment of overall breast cancer risk for women with a genetic predisposition, suggesting that the causality between MDD and breast cancer risk is robust and it was unlikely caused by confounders.

There were several advantages for the current two‐sample MR studies, first, because alleles follow the principle of random distribution when forming gametes during meiosis, the causal relationship between genotype and disease in MR studies will not be distorted by confounding factors, which is the main limitation of traditional observation studies. Second, it is much easier to use aggregated level data of public GWAS genetic consortia data to assess the causality relationships between MDD and breast cancer risk rather than the prospective cohorts studies or RCTs. The two‐sample MR study performed here required less time and expenses compare with common epidemiological studies. However, several limitations in the current studies need to be acknowledged in current study. First, the genetic IVs used in MR analysis are usually weak instruments, and only a small proportion of exposure variance was identified by the genetic IVs. The non‐causal connection between MDD and breast cancer in the stratification studies may be due to the low statistical power. Second, other confounding factors (such as population stratification and exposure time) may also affect the results. Third, because this study relies on aggregated GWAS data from people of European descent, the promotion of this result should be validated in other races.

In conclusion, the current MR analysis showed that MDD was causally associated with the increase of overall breast cancer. This result support the view that early intervention should be conducted on the women with MDD to reduce the risk of breast cancer; however, the results need to be confirmed using other study designs, including prospective cohort studies and large‐scale intervention trials.

## AUTHOR CONTRIBUTIONS

QR, FL, SG, and PC designed, researched, and revised the manuscript strictly. QR, FL, and PC analyzed the data set and wrote the manuscript. All authors approved the final version to be published.

## FUNDING INFORMATION

This study was supported, in part, by research funding from the National Key R&D Program of China (Grant No. 2017YFC0907001), the National Natural Science Foundation of Shanghai (Grant No. 20ZR1434100), and the Shanghai Municipal Health System Important and Weak Discipline (Clinical Nutrition) Project (Grant No. 2019ZB0104).

## CONFLICT OF INTEREST

The authors declare that the research was conducted in the absence of any commercial or financial relationships that could be construed as a potential conflict of interest.

## Supporting information


Figures S1‐S8

Table S1

Table S2

Table S3

Table S4

## Data Availability

The data that support the findings of this study are openly available in the IEU GWAS database (https://gwas.mrcieu.ac.uk) that comprises the harmonised complete GWAS summary datasets, which can be queried using the R package of TwoSampleMR.
