# Supplementary material for: Major depression disorder may causally associate with the increased breast cancer risk: Evidence from two‐sample mendelian randomization analyses
Source: Cancer Med. 2022 Jul 19;12(2):1984–96. doi: 10.1002/cam4.5043 (PMC9883582; doi:10.1002/cam4.5043)
Supplement: Supplementary file 1 — Figures S1‐S8 [file CAM4-12-1984-s005.docx]

**Supplementary Figures**

**Supplementary Figure 1.** Scatter plot of the two-sample Mendelian randomization (MR) results regarding the relationship between the effect size estimates on MDD (x-axis) and the effect size estimates on overall breast cancer (y-axis). The slope of colored lines represents the estimated causal effect obtained using the inverse variance weighted (IVW) and weighted median methods, respectively.

**Supplementary Figure 2.** Forest plot of the leave-one-out analysis of the two-sample MR study between MDD and overall breast cancer risk. Each black dot and horizontal line represent the causal effect obtained by the Wald ratio method and 95% CI for individual IV, respectively. The red dots and horizontal lines at the bottom represent the overall causal effects (ORs) and 95% CI under the conventional IVW model, respectively. X-axis is shown in natural logarithmic scale.

**Supplementary Figure 3.** Scatter plot of the two-sample Mendelian randomization (MR) results regarding the relationship between the effect size estimates on MDD (x-axis) and the effect size estimates on ER+ breast cancer (y-axis). The slope of colored lines represents the estimated causal effect obtained using the inverse variance weighted (IVW) and weighted median methods, respectively.

**Supplementary Figure 4.** Forest plot of the two-sample MR analysis results between MDD and the risk of ER+ breast cancer using the conventional inverse variance weighted (IVW) and weighted median methods. Each black dot and horizontal line represent the causal effect obtained by the Wald ratio method and 95% CI for individual IV, respectively. The red dots and horizontal lines at the bottom represent the overall causal effects (ORs) obtained by different methods and 95% CI, respectively.

**Supplementary Figure 5.** Forest plot of the leave-one-out analysis of the two-sample MR study between MDD and ER+ breast cancer risk. Each black dot and horizontal line represent the causal effect obtained by the Wald ratio method and 95% CI for individual IV, respectively. The red dots and horizontal lines at the bottom represent the overall causal effects (ORs) and 95% CI under the conventional IVW model, respectively. X-axis is shown in natural logarithmic scale.

**Supplementary Figure 6.** Scatter plot of the two-sample Mendelian randomization (MR) results regarding the relationship between the effect size estimates on MDD (x-axis) and the effect size estimates on ER- breast cancer (y-axis). The slope of colored lines represents the estimated causal effect obtained using the inverse variance weighted (IVW), fixed-effects IVW and simple median methods, respectively.

**Supplementary Figure 7.** Forest plot of the two-sample MR analysis results between MDD and the risk of ER- breast cancer using the conventional inverse variance weighted (IVW) and weighted median methods. Each black dot and horizontal line represent the causal effect obtained by the Wald ratio method and 95% CI for individual IV, respectively. The red dots and horizontal lines at the bottom represent the overall causal effects (ORs) obtained by different methods and 95% CI, respectively.

**Supplementary Figure 8.** Forest plot of the leave-one-out analysis of the two-sample MR study between MDD and ER- breast cancer risk. Each black dot and horizontal line represent the causal effect obtained by the Wald ratio method and 95% CI for individual IV, respectively. The red dots and horizontal lines at the bottom represent the overall causal effects (ORs) and 95% CI under the conventional IVW model, respectively. X-axis is shown in natural logarithmic scale.
