# Supplementary material for: Major depression disorder may causally associate with the increased breast cancer risk: Evidence from two‐sample mendelian randomization analyses
Source: Cancer Med. 2022 Jul 19;12(2):1984–96. doi: 10.1002/cam4.5043 (PMC9883582; doi:10.1002/cam4.5043)
Supplement: Supplementary file 2 — Table S1 [file CAM4-12-1984-s004.docx]

**Table S1.** Detailed information about the datasets used in current Mendelian randomization study.

| **Exposure/Outcome** | **Consortium** | **Participants** | **IEU GWAS database ID** |
| --- | --- | --- | --- |
| Major depression disease | Psychiatric Genomics Consortium (PGC) | 807,553 individuals including 246,363 cases and 561,190 controls of European ancestry | ieu-b-102 |
| Breast cancer | Breast Cancer Association Consortium (BCAC) | 228,951 women including 122,977 breast cancer (69,501 ER+ and 21,468 ER- patients) cases and 105,974 controls | ieu-a-1126 (overall breast cancer);  ieu-a-1127 (ER+ breast cancer);  ieu-a-1128 (ER- breast cancer) |
| Smoking initiation | GWAS and Sequencing Consortium of Alcohol and Nicotine use (GSCAN) | 607,291 European-descent individuals | ieu-b-4877 |
| Alcoholic drinks per week | GWAS and Sequencing Consortium of Alcohol and Nicotine use (GSCAN) | 335,394 European-descent individuals | ieu-b-73 |
| Education attainment (years of schooling) | Social Science Genetic Association Consortium (SSGAC) | 293,723 European-descent individuals | ieu-a-1001 |
| Household income (before tax) | MRC-IEU UK Biobank | MRC-IEU UK Biobank GWAS of European ancestry with 397,751 response individuals | ukb-b-7408 |
| Age at menarche | Reproductive Genetics (ReproGen) Consortium | 182,416 females of European ancestry | ieu-a-1095 |
