# Supplementary material for: Major depression disorder may causally associate with the increased breast cancer risk: Evidence from two‐sample mendelian randomization analyses
Source: Cancer Med. 2022 Jul 19;12(2):1984–96. doi: 10.1002/cam4.5043 (PMC9883582; doi:10.1002/cam4.5043)
Supplement: Supplementary file 3 — Table S2 [file CAM4-12-1984-s003.docx]

**Table S2.** Summary-level data of the associations between 92 validated IVs and overall, ER+ and ER- breast cancer risk from the Breast Cancer Association Consortium (BCAC) database.

| SNP | Chr | Position | Effect allele | Other Allele | Overall breast cancer | | | ER+ breast cancer | | | ER- breast cancer | | |
| --- | --- | --- | --- | --- | --- | --- | --- | --- | --- | --- | --- | --- | --- |
|  |  |  |  |  | beta | se | P-value | beta | se | P-value | beta | se | P-value |
| rs1226412 | 2 | 157111313 | T | C | -3.00E-04 | 0.0078 | 0.9723 | -0.0064 | 0.0093 | 0.4873 | 0.0114 | 0.0141 | 0.4184 |
| rs2670139 | 9 | 126634255 | C | T | 0.0194 | 0.0071 | 0.00662293 | 0.0195 | 0.0085 | 0.0217801 | 0.0178 | 0.0129 | 0.1692 |
| rs3099439 | 5 | 87545318 | T | C | -0.0042 | 0.0062 | 0.4984 | 0.0052 | 0.0074 | 0.4805 | -0.0113 | 0.0113 | 0.3169 |
| rs60157091 | 5 | 61509655 | T | C | 0.001 | 0.0067 | 0.8862 | -0.0039 | 0.008 | 0.624001 | 0.0129 | 0.012 | 0.2851 |
| rs1568452 | 2 | 58012833 | T | C | -0.0117 | 0.0063 | 0.0653702 | -0.0132 | 0.0076 | 0.0798693 | -0.0157 | 0.0116 | 0.1758 |
| rs10789214 | 1 | 67146817 | T | C | 0.0128 | 0.0063 | 0.0417004 | 0.018 | 0.0075 | 0.01689 | 0.0129 | 0.0115 | 0.264 |
| rs113188507 | 1 | 80809636 | A | G | -0.0055 | 0.0075 | 0.4604 | -0.0116 | 0.0089 | 0.1952 | 0.0016 | 0.0135 | 0.9068 |
| rs1448938 | 11 | 30892824 | G | A | -0.0011 | 0.0067 | 0.8733 | 0.0046 | 0.008 | 0.566601 | 0.0078 | 0.0121 | 0.5211 |
| rs2187490 | 11 | 118713180 | G | T | 0.0288 | 0.0114 | 0.01181 | 0.0249 | 0.0137 | 0.06827 | 0.0067 | 0.021 | 0.749701 |
| rs12923444 | 16 | 21639710 | C | A | -0.0029 | 0.0064 | 0.6579 | -0.0033 | 0.0077 | 0.6687 | -0.0119 | 0.0119 | 0.317 |
| rs8037355 | 15 | 37643831 | T | C | -0.006 | 0.0064 | 0.3476 | -0.0082 | 0.0077 | 0.2833 | -0.0045 | 0.0116 | 0.6968 |
| rs1021363 | 10 | 106610839 | G | A | -0.0044 | 0.0065 | 0.4968 | -0.001 | 0.0078 | 0.8992 | -0.0222 | 0.0119 | 0.0613296 |
| rs61990288 | 14 | 42074726 | A | G | 0.0013 | 0.0063 | 0.8344 | -0.0013 | 0.0075 | 0.8654 | -0.0038 | 0.0115 | 0.743401 |
| rs9545360 | 13 | 80826373 | A | C | 0.012 | 0.0084 | 0.15 | 0.0018 | 0.01 | 0.8533 | 0.0252 | 0.0151 | 0.0960993 |
| rs10149470 | 14 | 104017953 | G | A | 0.0114 | 0.0062 | 0.0681303 | 0.0125 | 0.0074 | 0.0925806 | 0.0145 | 0.0114 | 0.2009 |
| rs3793577 | 9 | 23737627 | G | A | 0.0046 | 0.0069 | 0.5042 | 0.0102 | 0.0082 | 0.2147 | 0.0125 | 0.0124 | 0.3141 |
| rs45510091 | 4 | 123186393 | G | A | 0.0066 | 0.0144 | 0.647 | 0.0072 | 0.0172 | 0.6766 | -0.0205 | 0.0269 | 0.4464 |
| rs169235 | 1 | 181740924 | G | A | -0.0082 | 0.0072 | 0.2515 | -0.0078 | 0.0086 | 0.3641 | -0.002 | 0.0131 | 0.8798 |
| rs16887442 | 7 | 82936909 | C | T | -0.013 | 0.0062 | 0.0371501 | -0.0089 | 0.0074 | 0.2337 | -0.0142 | 0.0114 | 0.2126 |
| rs3823624 | 7 | 2110346 | C | T | -0.0076 | 0.0078 | 0.328 | 0.0041 | 0.0093 | 0.6566 | -0.0099 | 0.0141 | 0.4833 |
| rs1466887 | 1 | 37709328 | T | C | 0.01 | 0.0063 | 0.1144 | 0.0052 | 0.0075 | 0.4857 | 0.015 | 0.0115 | 0.191 |
| rs7030813 | 9 | 36999369 | T | C | -0.0152 | 0.007 | 0.0285299 | -0.0116 | 0.0083 | 0.1623 | -0.0152 | 0.0126 | 0.2252 |
| rs34653192 | 9 | 31124452 | C | G | -0.0105 | 0.0074 | 0.1571 | -0.0084 | 0.0089 | 0.3441 | -0.0034 | 0.0136 | 0.802 |
| rs4772087 | 13 | 99115041 | T | C | -0.0122 | 0.0065 | 0.0622702 | -0.0181 | 0.0078 | 0.0198399 | -0.0184 | 0.0119 | 0.1216 |
| rs10890020 | 1 | 73668836 | G | A | 0.0024 | 0.0064 | 0.710799 | -0.0035 | 0.0076 | 0.6446 | 0.0217 | 0.0117 | 0.0632995 |
| rs200949 | 6 | 27835435 | G | A | -0.0403 | 0.0102 | 7.36E-05 | -0.046 | 0.0123 | 0.0001934 | -0.0341 | 0.0188 | 0.0696803 |
| rs58104186 | 7 | 109099919 | A | G | -0.0071 | 0.0063 | 0.2552 | -0.0108 | 0.0074 | 0.1466 | 0.0075 | 0.0114 | 0.5131 |
| rs1982277 | 9 | 11513019 | C | T | 5.00E-04 | 0.0076 | 0.9514 | 0.0047 | 0.0091 | 0.6041 | -0.0301 | 0.0139 | 0.0309001 |
| rs33431 | 19 | 30939989 | T | C | 0.0036 | 0.0068 | 0.6 | 0.006 | 0.0082 | 0.4601 | 6.00E-04 | 0.0123 | 0.9584 |
| rs1956373 | 14 | 60141822 | T | G | -7.00E-04 | 0.0075 | 0.9212 | -0.0036 | 0.0089 | 0.6899 | 0.0039 | 0.0135 | 0.769899 |
| rs7837935 | 8 | 65562019 | G | T | 0.0115 | 0.0091 | 0.2085 | 0.0156 | 0.0109 | 0.1506 | -0.0145 | 0.0165 | 0.3818 |
| rs1890946 | 1 | 52342427 | C | T | 0.0033 | 0.0062 | 0.5962 | 0.0063 | 0.0074 | 0.3911 | 0.0195 | 0.0113 | 0.0847403 |
| rs10913112 | 1 | 175913828 | T | C | -0.0052 | 0.0065 | 0.4197 | -0.0041 | 0.0078 | 0.6005 | -0.013 | 0.012 | 0.2782 |
| rs6783233 | 3 | 117509984 | T | C | 0.0012 | 0.007 | 0.8661 | -0.0005 | 0.0084 | 0.9491 | -0.0096 | 0.0128 | 0.4506 |
| rs1095626 | 3 | 157977962 | C | T | 0.0171 | 0.0063 | 0.00654606 | 0.0193 | 0.0075 | 0.0099949 | 0.0206 | 0.0114 | 0.0710706 |
| rs7758630 | 6 | 101387304 | A | T | 0.0068 | 0.0064 | 0.2893 | 0.0123 | 0.0077 | 0.1093 | -0.0038 | 0.0117 | 0.746299 |
| rs7200826 | 16 | 13066833 | T | C | -8.00E-04 | 0.0075 | 0.9117 | -0.0083 | 0.0089 | 0.3515 | 0.0175 | 0.0135 | 0.1945 |
| rs5995992 | 22 | 41487218 | C | T | 0.0014 | 0.0069 | 0.8422 | 0.0067 | 0.0083 | 0.4182 | 0.0089 | 0.0126 | 0.479001 |
| rs62091461 | 18 | 52488672 | T | C | -0.0041 | 0.0078 | 0.6027 | 0.0022 | 0.0094 | 0.8109 | -0.017 | 0.0142 | 0.2339 |
| rs12966052 | 18 | 52751639 | C | G | -0.0078 | 0.008 | 0.3336 | -0.0046 | 0.0096 | 0.6323 | -0.0076 | 0.0147 | 0.6071 |
| rs61902811 | 11 | 113370758 | A | G | -0.0048 | 0.0067 | 0.4708 | -0.0136 | 0.008 | 0.0882409 | 0.0101 | 0.0121 | 0.4032 |
| rs62188629 | 2 | 208044470 | A | G | -0.0068 | 0.0068 | 0.3163 | -0.0114 | 0.0081 | 0.1572 | -0.0227 | 0.0123 | 0.0655904 |
| rs30266 | 5 | 103972357 | A | G | 0.0077 | 0.0066 | 0.2463 | 0.0122 | 0.0079 | 0.1241 | -0.0138 | 0.0121 | 0.2532 |
| rs1354115 | 9 | 2983774 | A | C | 0.0019 | 0.007 | 0.7822 | -0.0048 | 0.0084 | 0.564901 | -0.0014 | 0.0126 | 0.9112 |
| rs1002656 | 1 | 37192741 | T | C | -0.012 | 0.0076 | 0.116 | -0.0094 | 0.0091 | 0.3003 | -0.029 | 0.0136 | 0.0326498 |
| rs725616 | 6 | 147950422 | C | T | -0.0016 | 0.0065 | 0.8072 | -0.0013 | 0.0078 | 0.8639 | 6.00E-04 | 0.0119 | 0.9573 |
| rs1343605 | 13 | 53647048 | C | A | 0.0177 | 0.0066 | 0.00759207 | 0.0268 | 0.0079 | 0.0007272 | 0.014 | 0.012 | 0.244 |
| rs9592461 | 13 | 66941792 | G | A | 0.0031 | 0.0063 | 0.6214 | 0.0099 | 0.0075 | 0.1879 | 0.0059 | 0.0115 | 0.6059 |
| rs4346585 | 3 | 44736493 | C | T | -0.0146 | 0.0067 | 0.0288197 | -0.016 | 0.008 | 0.0457899 | 0.0071 | 0.0122 | 0.5606 |
| rs57344483 | 11 | 127022560 | G | A | 0.0123 | 0.0119 | 0.3001 | 0.0173 | 0.0142 | 0.2213 | 0.0221 | 0.0214 | 0.303 |
| rs12967855 | 18 | 35138245 | G | A | -0.0059 | 0.0069 | 0.3965 | -0.0062 | 0.0083 | 0.452101 | 0.0033 | 0.0126 | 0.7926 |
| rs7227069 | 18 | 50731802 | A | G | 0.0021 | 0.0062 | 0.7373 | 5.00E-04 | 0.0074 | 0.9439 | 0.0062 | 0.0113 | 0.582101 |
| rs2509805 | 11 | 57650796 | C | T | -0.0154 | 0.0069 | 0.0254501 | -0.0127 | 0.0082 | 0.1219 | -6.00E-04 | 0.0125 | 0.9624 |
| rs7241572 | 18 | 77580712 | A | G | -0.0067 | 0.0089 | 0.4472 | -0.015 | 0.0107 | 0.1592 | -4.00E-04 | 0.0159 | 0.9787 |
| rs34488670 | 15 | 47684936 | C | T | 0.0062 | 0.0075 | 0.4101 | 0.0116 | 0.009 | 0.1964 | 0.0064 | 0.0137 | 0.6392 |
| rs7198928 | 16 | 7666402 | C | T | 4.00E-04 | 0.0065 | 0.948 | -0.0064 | 0.0077 | 0.4079 | 0.0149 | 0.0117 | 0.2061 |
| rs1409379 | 13 | 31907741 | T | C | 0.0169 | 0.0075 | 0.0239299 | 0.0224 | 0.0089 | 0.01224 | 0.0264 | 0.0136 | 0.0527995 |
| rs1045430 | 14 | 75130235 | G | T | 0.0018 | 0.0063 | 0.7804 | -0.0023 | 0.0075 | 0.7634 | 0.0155 | 0.0115 | 0.175 |
| rs75581564 | 17 | 27363750 | A | G | 0.0038 | 0.0101 | 0.7045 | -0.0109 | 0.0121 | 0.3684 | -0.0045 | 0.0184 | 0.8063 |
| rs143186028 | 20 | 39997404 | T | G | 0.0237 | 0.0082 | 0.00380899 | 0.0305 | 0.0097 | 0.001726 | 0.0014 | 0.015 | 0.9249 |
| rs913930 | 9 | 120484009 | A | G | -0.0049 | 0.0068 | 0.4701 | -0.0091 | 0.0082 | 0.2671 | -0.0076 | 0.0124 | 0.5409 |
| rs12967143 | 18 | 53099012 | C | G | -0.0036 | 0.0068 | 0.5989 | -0.0036 | 0.0082 | 0.6585 | -5.00E-04 | 0.0125 | 0.969 |
| rs10061069 | 5 | 93071630 | C | G | -0.002 | 0.0078 | 0.7982 | -0.0045 | 0.0093 | 0.6293 | 0.0226 | 0.0142 | 0.1126 |
| rs59283172 | 9 | 25232978 | A | G | 0.0114 | 0.0103 | 0.269 | 0.0141 | 0.0123 | 0.2498 | 0.0323 | 0.0188 | 0.0853906 |
| rs7117514 | 11 | 70544937 | G | A | 0.0094 | 0.0064 | 0.1432 | 0.0115 | 0.0077 | 0.1339 | -0.004 | 0.0117 | 0.7326 |
| rs58621819 | 11 | 65314830 | T | A | 0.0191 | 0.008 | 0.0172401 | 0.0143 | 0.0096 | 0.1361 | 0.0167 | 0.0146 | 0.2518 |
| rs7932640 | 11 | 88744425 | C | T | 0.0032 | 0.0062 | 0.6069 | 0.0068 | 0.0074 | 0.3607 | -0.0014 | 0.0114 | 0.9029 |
| rs34937911 | 4 | 42110353 | C | T | -0.0057 | 0.0103 | 0.5779 | -0.0068 | 0.0124 | 0.5837 | -0.0089 | 0.0187 | 0.631499 |
| rs301799 | 1 | 8489302 | T | C | -0.0081 | 0.0065 | 0.2152 | -0.0079 | 0.0078 | 0.3109 | 0.0084 | 0.0119 | 0.4785 |
| rs7685686 | 4 | 3207142 | G | A | -0.0074 | 0.0063 | 0.2406 | -0.0038 | 0.0075 | 0.609901 | -0.0024 | 0.0114 | 0.8356 |
| rs9363467 | 6 | 66565703 | C | T | -0.0119 | 0.0064 | 0.0602199 | -0.0032 | 0.0076 | 0.677699 | -0.014 | 0.0116 | 0.2276 |
| rs7585722 | 2 | 86819128 | C | T | -0.0038 | 0.009 | 0.6736 | -0.0088 | 0.0107 | 0.4105 | -6.00E-04 | 0.0162 | 0.9702 |
| rs17641524 | 1 | 197704717 | T | C | 0.002 | 0.0081 | 0.8085 | 0.001 | 0.0097 | 0.9161 | 0.0101 | 0.0147 | 0.4927 |
| rs72710803 | 1 | 177428018 | C | A | 0.0052 | 0.0113 | 0.644899 | 0.0088 | 0.0134 | 0.5136 | 0.0195 | 0.0205 | 0.3417 |
| rs11135349 | 5 | 164523472 | C | A | -0.0063 | 0.0065 | 0.3323 | -0.0058 | 0.0077 | 0.4494 | 0.0048 | 0.0118 | 0.681999 |
| rs7624336 | 3 | 53244151 | T | G | 0.0169 | 0.0081 | 0.0370502 | 0.0257 | 0.0097 | 0.0082181 | 0.0219 | 0.0146 | 0.1349 |
| rs141954845 | 3 | 61192911 | A | G | -0.0032 | 0.0065 | 0.6175 | -0.0016 | 0.0077 | 0.8326 | 0.0037 | 0.0117 | 0.7552 |
| rs13084037 | 3 | 49214066 | A | G | -0.0165 | 0.0075 | 0.0276198 | -0.0101 | 0.009 | 0.2608 | -0.0231 | 0.0137 | 0.0920704 |
| rs67436663 | 8 | 71347626 | C | G | -0.0019 | 0.0073 | 0.7966 | 0.0062 | 0.0087 | 0.4751 | -0.0057 | 0.0133 | 0.6687 |
| rs7659414 | 4 | 177350956 | C | A | -5.00E-04 | 0.0065 | 0.9439 | -0.0087 | 0.0078 | 0.2664 | 0.0114 | 0.0118 | 0.3373 |
| rs35553410 | 4 | 131237381 | C | T | 0.0013 | 0.0074 | 0.8578 | 0.0037 | 0.0089 | 0.679499 | -0.0018 | 0.0134 | 0.8925 |
| rs2568958 | 1 | 72765116 | A | G | -0.0188 | 0.0064 | 0.003059 | -0.0189 | 0.0076 | 0.01253 | -0.016 | 0.0116 | 0.169 |
| rs11579246 | 1 | 50559162 | G | A | 0.0015 | 0.0102 | 0.8859 | -0.016 | 0.0122 | 0.1893 | 0.0063 | 0.0187 | 0.737099 |
| rs7193263 | 16 | 6315880 | A | G | -0.0027 | 0.0069 | 0.6962 | -0.0016 | 0.0082 | 0.8454 | 0.0035 | 0.0125 | 0.780001 |
| rs56887639 | 16 | 13755530 | G | A | -0.0038 | 0.0071 | 0.5952 | 0.0052 | 0.0084 | 0.5384 | -0.0278 | 0.0129 | 0.0307001 |
| rs997934 | 10 | 1795194 | C | T | 0.0068 | 0.0072 | 0.3437 | 0.0036 | 0.0086 | 0.680201 | -0.0053 | 0.0129 | 0.679401 |
| rs10817969 | 9 | 119731045 | G | T | -0.0039 | 0.0071 | 0.576901 | 0.0012 | 0.0084 | 0.8882 | -0.0068 | 0.0128 | 0.5936 |
| rs2043539 | 7 | 12253880 | A | G | -2.00E-04 | 0.0063 | 0.9763 | -0.0021 | 0.0075 | 0.777401 | -0.0073 | 0.0114 | 0.5214 |
| rs1152578 | 14 | 64697037 | C | T | 0.0035 | 0.0063 | 0.5772 | 0.0059 | 0.0075 | 0.4287 | 0.006 | 0.0114 | 0.5968 |
| rs198457 | 11 | 61471678 | T | C | -0.004 | 0.0084 | 0.6316 | -0.0115 | 0.01 | 0.2503 | 0.0222 | 0.0152 | 0.1435 |
| rs7807677 | 7 | 117502574 | T | C | 0.0023 | 0.0064 | 0.7226 | 0.0095 | 0.0076 | 0.2143 | -0.0121 | 0.0116 | 0.2973 |
| rs12624433 | 20 | 44680853 | A | G | -0.0126 | 0.0073 | 0.0824594 | -0.019 | 0.0087 | 0.0277703 | 2.00E-04 | 0.0132 | 0.9869 |

**Abbreviations:** ER, estrogen receptor; SNP, single nucleotide polymorphism.
