# Supplementary material for: Major depression disorder may causally associate with the increased breast cancer risk: Evidence from two‐sample mendelian randomization analyses
Source: Cancer Med. 2022 Jul 19;12(2):1984–96. doi: 10.1002/cam4.5043 (PMC9883582; doi:10.1002/cam4.5043)
Supplement: Supplementary file 4 — Table S3 [file CAM4-12-1984-s001.docx]

**Table S3.** Sample size and power calculations in Mendelian randomization study of MDD and risks of breast cancer for the 92 genetic instrumental variables.

| **Outcome** | **Sample Size** | **Proportion of cases** | **Power of estimated Odd Ratio** | | |  | **Post hoc test** | |
| --- | --- | --- | --- | --- | --- | --- | --- | --- |
|  |  |  | **OR = 1.05** | **OR = 1.10** | **OR = 1.15** |  | **OR** | **Power** |
| Overall | 228,951 (122,977/105,974) | 0.54 | 0.44 | 0.94 | 1 |  | 1.090 | 0.89 |
| ER+ | 175,475 (69,501/105,974) | 0.4 | 0.35 | 0.87 | 1 |  | 1.059 | 0.46 |
| ER- | 127,442 (21,468/105,974) | 0.17 | 0.18 | 0.54 | 0.87 |  | 1.101 | 0.55 |

**Abbreviations:** ER, estrogen receptor; OR, odds ratio.
