# Supplementary material for: Major depression disorder may causally associate with the increased breast cancer risk: Evidence from two‐sample mendelian randomization analyses
Source: Cancer Med. 2022 Jul 19;12(2):1984–96. doi: 10.1002/cam4.5043 (PMC9883582; doi:10.1002/cam4.5043)
Supplement: Supplementary file 5 — Table S4 [file CAM4-12-1984-s002.docx]

**Table S4.** Summary-level data of IVs used in multivariable MR studies that independently associated with smoking, alcohol intake, educational attainment (years of schooling), household income (before tax) and age at menarche.

| SNP | Chr | Position | beta | se | Sample Size | P-value | EAF | Effect allele | Other allele | Outcome | ID |
| --- | --- | --- | --- | --- | --- | --- | --- | --- | --- | --- | --- |
| rs9401770 | 6 | 98748008 | 0.0277307 | 0.003986 | 632802 | 3.47E-12 | 0.273 | A | G | smoking initiation | ieu-b-4877 |
| rs222449 | 6 | 52916062 | -0.0253208 | 0.004428 | 632802 | 1.08E-08 | 0.793 | T | A | smoking initiation | ieu-b-4877 |
| rs12025237 | 1 | 154205120 | -0.0329992 | 0.0053392 | 632802 | 6.52E-10 | 0.124 | C | A | smoking initiation | ieu-b-4877 |
| rs12042107 | 1 | 91196176 | -0.0222834 | 0.0035682 | 632802 | 4.22E-10 | 0.527 | C | T | smoking initiation | ieu-b-4877 |
| rs13030994 | 2 | 146143090 | 0.0360925 | 0.0035563 | 632802 | 3.56E-24 | 0.485 | A | G | smoking initiation | ieu-b-4877 |
| rs10001365 | 4 | 147797214 | -0.0249918 | 0.0036416 | 632802 | 6.65E-12 | 0.405 | A | G | smoking initiation | ieu-b-4877 |
| rs10260968 | 7 | 1889773 | -0.0203218 | 0.0036094 | 632802 | 1.75E-08 | 0.597 | A | G | smoking initiation | ieu-b-4877 |
| rs4781977 | 16 | 17572674 | -0.0238668 | 0.0043647 | 632802 | 4.54E-08 | 0.205 | C | T | smoking initiation | ieu-b-4877 |
| rs1050847 | 16 | 87443734 | -0.0216231 | 0.0035889 | 632802 | 1.67E-09 | 0.505 | T | C | smoking initiation | ieu-b-4877 |
| rs11872397 | 18 | 72535282 | -0.0247725 | 0.0040948 | 632802 | 1.43E-09 | 0.252 | A | G | smoking initiation | ieu-b-4877 |
| rs2186122 | 1 | 66470206 | 0.0260573 | 0.003586 | 632802 | 3.61E-13 | 0.561 | T | A | smoking initiation | ieu-b-4877 |
| rs6788098 | 3 | 85624131 | -0.0313461 | 0.0036891 | 632802 | 1.91E-17 | 0.623 | T | A | smoking initiation | ieu-b-4877 |
| rs66680800 | 3 | 85985324 | -0.0202716 | 0.0036527 | 632802 | 2.83E-08 | 0.397 | T | G | smoking initiation | ieu-b-4877 |
| rs12356821 | 10 | 104563808 | 0.03937 | 0.0050491 | 632802 | 6.27E-15 | 0.14 | C | G | smoking initiation | ieu-b-4877 |
| rs6508144 | 18 | 50026142 | -0.0206935 | 0.003586 | 632802 | 7.97E-09 | 0.563 | G | C | smoking initiation | ieu-b-4877 |
| rs10159545 | 10 | 21766969 | 0.0262501 | 0.0037273 | 632802 | 1.84E-12 | 0.375 | G | C | smoking initiation | ieu-b-4877 |
| rs6265 | 11 | 27679916 | -0.0317863 | 0.0045784 | 632802 | 3.77E-12 | 0.203 | T | C | smoking initiation | ieu-b-4877 |
| rs4759228 | 12 | 56508409 | -0.0216913 | 0.0039341 | 632802 | 3.58E-08 | 0.27 | C | G | smoking initiation | ieu-b-4877 |
| rs7585579 | 2 | 60024857 | 0.0223996 | 0.0037281 | 632802 | 1.88E-09 | 0.505 | G | C | smoking initiation | ieu-b-4877 |
| rs266047 | 2 | 104088751 | -0.0305098 | 0.0037386 | 632802 | 3.36E-16 | 0.529 | A | G | smoking initiation | ieu-b-4877 |
| rs6728726 | 2 | 623976 | 0.0354486 | 0.0047328 | 632802 | 6.73E-14 | 0.829 | C | T | smoking initiation | ieu-b-4877 |
| rs4674993 | 2 | 226332033 | -0.0252122 | 0.0044362 | 632802 | 1.32E-08 | 0.207 | G | A | smoking initiation | ieu-b-4877 |
| rs10498846 | 6 | 67405337 | 0.0206103 | 0.0035556 | 632802 | 6.62E-09 | 0.473 | T | C | smoking initiation | ieu-b-4877 |
| rs12333760 | 7 | 99185406 | -0.0290467 | 0.0048013 | 632802 | 1.44E-09 | 0.204 | C | T | smoking initiation | ieu-b-4877 |
| rs1971318 | 12 | 121389500 | 0.0285074 | 0.0049253 | 632802 | 7.06E-09 | 0.141 | T | C | smoking initiation | ieu-b-4877 |
| rs1555445 | 20 | 31175258 | 0.0225548 | 0.0038234 | 632802 | 3.65E-09 | 0.337 | T | A | smoking initiation | ieu-b-4877 |
| rs7929518 | 11 | 85980958 | 0.0242377 | 0.0042847 | 632802 | 1.56E-08 | 0.765 | G | A | smoking initiation | ieu-b-4877 |
| rs72789632 | 5 | 106834363 | -0.0328856 | 0.0052863 | 632802 | 5.02E-10 | 0.12 | T | C | smoking initiation | ieu-b-4877 |
| rs4044321 | 5 | 166989513 | -0.0278417 | 0.0037106 | 632802 | 6.08E-14 | 0.642 | G | A | smoking initiation | ieu-b-4877 |
| rs3904512 | 13 | 38357471 | -0.0211589 | 0.0035765 | 632802 | 3.23E-09 | 0.429 | A | G | smoking initiation | ieu-b-4877 |
| rs993700 | 4 | 67825894 | -0.025928 | 0.0042916 | 632802 | 1.53E-09 | 0.766 | C | T | smoking initiation | ieu-b-4877 |
| rs1899896 | 8 | 93201036 | 0.0264481 | 0.0038869 | 632802 | 1.04E-11 | 0.286 | T | C | smoking initiation | ieu-b-4877 |
| rs2140114 | 7 | 3407568 | -0.0232591 | 0.003734 | 632802 | 4.70E-10 | 0.518 | T | C | smoking initiation | ieu-b-4877 |
| rs3800227 | 6 | 108994161 | 0.0228121 | 0.0040581 | 632802 | 1.93E-08 | 0.701 | G | A | smoking initiation | ieu-b-4877 |
| rs6433897 | 2 | 182034448 | 0.0224483 | 0.0040581 | 632802 | 3.16E-08 | 0.754 | C | T | smoking initiation | ieu-b-4877 |
| rs12632110 | 3 | 50224225 | -0.0233768 | 0.0037529 | 632802 | 4.78E-10 | 0.647 | G | A | smoking initiation | ieu-b-4877 |
| rs1385108 | 5 | 154839646 | 0.0246617 | 0.0041567 | 632802 | 3.00E-09 | 0.239 | T | C | smoking initiation | ieu-b-4877 |
| rs2631024 | 8 | 91995577 | -0.0229645 | 0.0040282 | 632802 | 1.18E-08 | 0.737 | G | A | smoking initiation | ieu-b-4877 |
| rs1160685 | 4 | 94052854 | 0.0207724 | 0.0035889 | 632802 | 7.20E-09 | 0.478 | G | C | smoking initiation | ieu-b-4877 |
| rs4236259 | 7 | 1708080 | -0.0247689 | 0.0035566 | 632802 | 3.35E-12 | 0.499 | G | T | smoking initiation | ieu-b-4877 |
| rs2378662 | 9 | 86707289 | 0.0209482 | 0.0035665 | 632802 | 4.16E-09 | 0.556 | A | G | smoking initiation | ieu-b-4877 |
| rs117143374 | 21 | 40555561 | 0.0292897 | 0.0052691 | 632802 | 2.76E-08 | 0.12 | C | T | smoking initiation | ieu-b-4877 |
| rs9423279 | 10 | 125680419 | -0.0205132 | 0.0037083 | 632802 | 3.21E-08 | 0.641 | G | C | smoking initiation | ieu-b-4877 |
| rs3001723 | 1 | 44037685 | 0.0335118 | 0.0038983 | 632802 | 8.12E-18 | 0.321 | A | G | smoking initiation | ieu-b-4877 |
| rs2046850 | 1 | 210304319 | -0.0248139 | 0.0044784 | 632802 | 3.03E-08 | 0.187 | T | C | smoking initiation | ieu-b-4877 |
| rs11712680 | 3 | 75009019 | -0.0270476 | 0.0045784 | 632802 | 3.51E-09 | 0.174 | C | A | smoking initiation | ieu-b-4877 |
| rs7921378 | 10 | 63674885 | -0.0254601 | 0.0035582 | 632802 | 8.26E-13 | 0.463 | C | G | smoking initiation | ieu-b-4877 |
| rs4543592 | 9 | 3014254 | 0.0219314 | 0.0035624 | 632802 | 7.46E-10 | 0.468 | C | T | smoking initiation | ieu-b-4877 |
| rs962625 | 4 | 28473524 | 0.0237181 | 0.004038 | 632802 | 4.37E-09 | 0.24 | G | A | smoking initiation | ieu-b-4877 |
| rs12112638 | 7 | 69735251 | -0.024526 | 0.004043 | 632802 | 1.34E-09 | 0.275 | G | A | smoking initiation | ieu-b-4877 |
| rs3801289 | 7 | 96638267 | -0.0220618 | 0.0037398 | 632802 | 3.74E-09 | 0.351 | C | A | smoking initiation | ieu-b-4877 |
| rs10279261 | 7 | 133589846 | -0.0214194 | 0.0036626 | 632802 | 5.00E-09 | 0.619 | A | G | smoking initiation | ieu-b-4877 |
| rs240963 | 6 | 111644332 | -0.0410444 | 0.0048371 | 632802 | 2.16E-17 | 0.836 | C | T | smoking initiation | ieu-b-4877 |
| rs12545053 | 8 | 65073605 | 0.0202808 | 0.0036367 | 632802 | 2.43E-08 | 0.397 | G | A | smoking initiation | ieu-b-4877 |
| rs10905461 | 10 | 8803551 | -0.0239554 | 0.0041451 | 632802 | 7.35E-09 | 0.718 | C | T | smoking initiation | ieu-b-4877 |
| rs7938812 | 11 | 112911004 | 0.0437914 | 0.0036367 | 632802 | 2.71E-33 | 0.424 | G | T | smoking initiation | ieu-b-4877 |
| rs11057005 | 12 | 16748721 | -0.0209298 | 0.0035789 | 632802 | 4.85E-09 | 0.43 | G | A | smoking initiation | ieu-b-4877 |
| rs7322872 | 13 | 100548329 | -0.0255713 | 0.0043347 | 632802 | 3.58E-09 | 0.782 | T | C | smoking initiation | ieu-b-4877 |
| rs1869243 | 3 | 5724536 | 0.0197411 | 0.0035629 | 632802 | 2.97E-08 | 0.481 | C | T | smoking initiation | ieu-b-4877 |
| rs578584 | 2 | 45143175 | 0.0286801 | 0.0035963 | 632802 | 1.50E-15 | 0.605 | T | A | smoking initiation | ieu-b-4877 |
| rs11078713 | 17 | 7795972 | -0.0201721 | 0.0036056 | 632802 | 2.23E-08 | 0.454 | G | A | smoking initiation | ieu-b-4877 |
| rs7197072 | 16 | 717085 | -0.0247672 | 0.0041686 | 632802 | 2.77E-09 | 0.238 | T | C | smoking initiation | ieu-b-4877 |
| rs12441907 | 15 | 83922387 | -0.0292051 | 0.0045226 | 632802 | 1.06E-10 | 0.186 | A | C | smoking initiation | ieu-b-4877 |
| rs1435741 | 15 | 47935843 | 0.0294151 | 0.003591 | 632802 | 2.64E-16 | 0.425 | A | G | smoking initiation | ieu-b-4877 |
| rs72896886 | 18 | 42632652 | -0.0268885 | 0.0048371 | 632802 | 2.75E-08 | 0.144 | C | G | smoking initiation | ieu-b-4877 |
| rs6893752 | 5 | 60374912 | -0.0240995 | 0.0040736 | 632802 | 3.25E-09 | 0.766 | G | A | smoking initiation | ieu-b-4877 |
| rs76214862 | 14 | 29500130 | -0.0249903 | 0.0045475 | 632802 | 3.99E-08 | 0.202 | C | A | smoking initiation | ieu-b-4877 |
| rs7969559 | 12 | 69655167 | -0.0243756 | 0.0039595 | 632802 | 7.31E-10 | 0.688 | G | A | smoking initiation | ieu-b-4877 |
| rs12186738 | 5 | 103816655 | -0.0332644 | 0.0050205 | 632802 | 3.42E-11 | 0.154 | T | G | smoking initiation | ieu-b-4877 |
| rs2050586 | 1 | 87905828 | -0.0205467 | 0.0037083 | 632802 | 3.00E-08 | 0.355 | C | G | smoking initiation | ieu-b-4877 |
| rs35702515 | 2 | 137542847 | 0.0252442 | 0.0042309 | 632802 | 2.43E-09 | 0.162 | T | G | smoking initiation | ieu-b-4877 |
| rs1445649 | 2 | 155682556 | 0.0239932 | 0.0035648 | 632802 | 1.68E-11 | 0.525 | C | T | smoking initiation | ieu-b-4877 |
| rs12474587 | 2 | 162802993 | 0.0276329 | 0.0035823 | 632802 | 1.25E-14 | 0.404 | T | G | smoking initiation | ieu-b-4877 |
| rs13261666 | 8 | 59814666 | -0.0268946 | 0.003556 | 632802 | 3.90E-14 | 0.522 | T | G | smoking initiation | ieu-b-4877 |
| rs10114490 | 9 | 11070165 | -0.0255148 | 0.0045317 | 632802 | 1.81E-08 | 0.198 | A | G | smoking initiation | ieu-b-4877 |
| rs1565735 | 8 | 27426077 | -0.037618 | 0.0044613 | 632802 | 3.42E-17 | 0.212 | A | T | smoking initiation | ieu-b-4877 |
| rs4523689 | 11 | 7950797 | -0.0206091 | 0.0036432 | 632802 | 1.55E-08 | 0.408 | G | A | smoking initiation | ieu-b-4877 |
| rs13145728 | 4 | 140927812 | -0.0232512 | 0.0036626 | 632802 | 2.14E-10 | 0.358 | C | G | smoking initiation | ieu-b-4877 |
| rs2107300 | 2 | 200937901 | -0.027201 | 0.0049253 | 632802 | 3.27E-08 | 0.845 | G | C | smoking initiation | ieu-b-4877 |
| rs6669839 | 1 | 50625979 | 0.0260041 | 0.0043955 | 632802 | 3.36E-09 | 0.204 | T | C | smoking initiation | ieu-b-4877 |
| rs1154693 | 3 | 117804154 | 0.0326217 | 0.0049123 | 632802 | 3.12E-11 | 0.856 | G | A | smoking initiation | ieu-b-4877 |
| rs76608582 | 19 | 4474725 | -0.0495575 | 0.0082596 | 632802 | 1.94E-09 | 0.0389 | A | C | smoking initiation | ieu-b-4877 |
| rs11658881 | 17 | 2072949 | 0.0201357 | 0.0036107 | 632802 | 2.43E-08 | 0.418 | G | A | smoking initiation | ieu-b-4877 |
| rs4785836 | 16 | 65604652 | -0.0204704 | 0.0036589 | 632802 | 2.26E-08 | 0.398 | C | T | smoking initiation | ieu-b-4877 |
| rs7224742 | 17 | 30657058 | -0.0207099 | 0.0036553 | 632802 | 1.43E-08 | 0.595 | T | C | smoking initiation | ieu-b-4877 |
| rs134529 | 22 | 28781758 | -0.019984 | 0.0036608 | 632802 | 4.85E-08 | 0.349 | C | T | smoking initiation | ieu-b-4877 |
| rs331939 | 4 | 143654889 | -0.0119016 | 0.0020292 | 536700 | 4.50E-09 | 0.339 | A | G | Alcoholic drinks per week | ieu-b-73 |
| rs1229984 | 4 | 100239319 | 0.188115 | 0.0061785 | 514602 | 1.60E-203 | 0.953 | C | T | Alcoholic drinks per week | ieu-b-73 |
| rs78234152 | 4 | 100279889 | 0.0276538 | 0.0030708 | 534076 | 2.18E-19 | 0.0986 | A | G | Alcoholic drinks per week | ieu-b-73 |
| rs28712821 | 4 | 39413780 | 0.0283339 | 0.0019741 | 525616 | 1.10E-46 | 0.594 | A | G | Alcoholic drinks per week | ieu-b-73 |
| rs16854020 | 4 | 42117559 | 0.018083 | 0.0029068 | 533501 | 4.82E-10 | 0.127 | A | G | Alcoholic drinks per week | ieu-b-73 |
| rs55932213 | 9 | 108755622 | 0.0124767 | 0.002216 | 515956 | 1.80E-08 | 0.701 | G | A | Alcoholic drinks per week | ieu-b-73 |
| rs28601761 | 8 | 126500031 | 0.0112989 | 0.0019551 | 523035 | 7.60E-09 | 0.405 | G | C | Alcoholic drinks per week | ieu-b-73 |
| rs10085696 | 7 | 69783020 | -0.0160517 | 0.0024947 | 535056 | 1.24E-10 | 0.201 | G | A | Alcoholic drinks per week | ieu-b-73 |
| rs2049045 | 11 | 27694241 | -0.0137683 | 0.0025054 | 532726 | 3.97E-08 | 0.189 | C | G | Alcoholic drinks per week | ieu-b-73 |
| rs28680958 | 1 | 173848808 | -0.0135848 | 0.0023684 | 532042 | 9.78E-09 | 0.23 | A | G | Alcoholic drinks per week | ieu-b-73 |
| rs1387766 | 12 | 92081800 | -0.010827 | 0.0019834 | 535067 | 4.79E-08 | 0.622 | A | G | Alcoholic drinks per week | ieu-b-73 |
| rs962961 | 14 | 57281154 | -0.0121885 | 0.0020515 | 533853 | 2.78E-09 | 0.329 | T | C | Alcoholic drinks per week | ieu-b-73 |
| rs11860773 | 16 | 73912503 | -0.0150051 | 0.0024438 | 515422 | 8.35E-10 | 0.176 | C | T | Alcoholic drinks per week | ieu-b-73 |
| rs28732378 | 3 | 85403892 | -0.0167274 | 0.0021908 | 530009 | 2.24E-14 | 0.729 | G | A | Alcoholic drinks per week | ieu-b-73 |
| rs6106989 | 20 | 25027630 | 0.0108994 | 0.0019834 | 520623 | 3.81E-08 | 0.628 | A | G | Alcoholic drinks per week | ieu-b-73 |
| rs13332432 | 16 | 85721809 | 0.014005 | 0.0021407 | 516946 | 5.94E-11 | 0.296 | G | C | Alcoholic drinks per week | ieu-b-73 |
| rs34121753 | 17 | 7733833 | 0.0110694 | 0.0019507 | 518183 | 1.39E-08 | 0.532 | G | A | Alcoholic drinks per week | ieu-b-73 |
| rs676388 | 19 | 49211969 | 0.0150976 | 0.0019315 | 531996 | 5.49E-15 | 0.494 | C | T | Alcoholic drinks per week | ieu-b-73 |
| rs75120545 | 2 | 44271496 | -0.0327862 | 0.0056731 | 474779 | 7.59E-09 | 0.022 | T | C | Alcoholic drinks per week | ieu-b-73 |
| rs494904 | 2 | 45141180 | 0.0150848 | 0.0019606 | 531073 | 1.41E-14 | 0.429 | C | T | Alcoholic drinks per week | ieu-b-73 |
| rs2299409 | 7 | 103812171 | -0.0105546 | 0.0019335 | 534249 | 4.80E-08 | 0.493 | A | G | Alcoholic drinks per week | ieu-b-73 |
| rs6739804 | 2 | 63269604 | -0.0129688 | 0.002082 | 532202 | 4.72E-10 | 0.66 | C | T | Alcoholic drinks per week | ieu-b-73 |
| rs28929474 | 14 | 94844947 | -0.0476809 | 0.0071397 | 528430 | 2.39E-11 | 0.0154 | T | C | Alcoholic drinks per week | ieu-b-73 |
| rs4752999 | 11 | 47428565 | -0.0145643 | 0.0020701 | 529379 | 2.03E-12 | 0.321 | T | C | Alcoholic drinks per week | ieu-b-73 |
| rs55872084 | 5 | 155902003 | 0.0127314 | 0.0022684 | 529669 | 1.98E-08 | 0.218 | T | G | Alcoholic drinks per week | ieu-b-73 |
| rs13107325 | 4 | 103188709 | -0.0364548 | 0.0039129 | 528164 | 1.23E-20 | 0.0654 | T | C | Alcoholic drinks per week | ieu-b-73 |
| rs79616692 | 16 | 72338507 | 0.0188085 | 0.0031523 | 529527 | 2.38E-09 | 0.11 | C | G | Alcoholic drinks per week | ieu-b-73 |
| rs153106 | 16 | 28526897 | -0.0136118 | 0.0019586 | 531637 | 3.63E-12 | 0.409 | C | T | Alcoholic drinks per week | ieu-b-73 |
| rs1260326 | 2 | 27730940 | 0.023812 | 0.0019843 | 532340 | 3.33E-33 | 0.595 | C | T | Alcoholic drinks per week | ieu-b-73 |
| rs6969458 | 7 | 153489725 | 0.0127063 | 0.0019355 | 509646 | 5.20E-11 | 0.459 | A | G | Alcoholic drinks per week | ieu-b-73 |
| rs17542254 | 11 | 113655696 | 0.0131418 | 0.0021461 | 533884 | 8.96E-10 | 0.251 | G | A | Alcoholic drinks per week | ieu-b-73 |
| rs76640332 | 17 | 44189858 | -0.0210081 | 0.0023894 | 514949 | 1.47E-18 | 0.204 | A | G | Alcoholic drinks per week | ieu-b-73 |
| rs12987662 | 2 | 100821548 | 0.022 | 0.003 | 293723 | 3.25E-18 | 0.3787 | A | C | Years of schooling | ieu-a-1001 |
| rs111321694 | 11 | 110950386 | -0.018 | 0.003 | 293723 | 4.23E-08 | 0.1828 | T | C | Years of schooling | ieu-a-1001 |
| rs10772644 | 12 | 13417617 | 0.021 | 0.004 | 293723 | 4.11E-08 | 0.8713 | C | G | Years of schooling | ieu-a-1001 |
| rs10831912 | 11 | 12856414 | 0.015 | 0.003 | 293723 | 1.23E-08 | 0.597 | C | T | Years of schooling | ieu-a-1001 |
| rs10483349 | 14 | 29629456 | 0.019 | 0.003 | 293723 | 3.02E-09 | 0.1698 | G | A | Years of schooling | ieu-a-1001 |
| rs4863692 | 4 | 140764124 | 0.018 | 0.003 | 293723 | 3.80E-12 | 0.334 | T | G | Years of schooling | ieu-a-1001 |
| rs12534506 | 7 | 92662327 | 0.015 | 0.003 | 293723 | 7.95E-09 | 0.5466 | T | A | Years of schooling | ieu-a-1001 |
| rs1106761 | 8 | 142619234 | -0.017 | 0.003 | 293723 | 4.08E-11 | 0.3601 | A | G | Years of schooling | ieu-a-1001 |
| rs4244613 | 8 | 145741765 | -0.014 | 0.003 | 293723 | 9.94E-09 | 0.416 | A | G | Years of schooling | ieu-a-1001 |
| rs8049439 | 16 | 28837515 | -0.015 | 0.003 | 293723 | 2.69E-09 | 0.3451 | C | T | Years of schooling | ieu-a-1001 |
| rs4478846 | 1 | 98396847 | 0.018 | 0.003 | 293723 | 1.93E-08 | 0.8526 | T | C | Years of schooling | ieu-a-1001 |
| rs7599488 | 2 | 60718347 | -0.017 | 0.002 | 293723 | 2.05E-11 | 0.4254 | T | C | Years of schooling | ieu-a-1001 |
| rs4493682 | 5 | 45188024 | 0.019 | 0.003 | 293723 | 2.27E-08 | 0.2034 | C | G | Years of schooling | ieu-a-1001 |
| rs4974424 | 3 | 127148720 | 0.019 | 0.003 | 293723 | 2.64E-08 | 0.1735 | G | A | Years of schooling | ieu-a-1001 |
| rs7029201 | 9 | 23358081 | 0.025 | 0.003 | 293723 | 6.13E-23 | 0.4235 | A | G | Years of schooling | ieu-a-1001 |
| rs7033137 | 9 | 72055158 | -0.016 | 0.003 | 293723 | 2.14E-08 | 0.2257 | G | C | Years of schooling | ieu-a-1001 |
| rs1424580 | 7 | 133154426 | 0.018 | 0.003 | 293723 | 9.58E-09 | 0.8004 | T | C | Years of schooling | ieu-a-1001 |
| rs11191193 | 10 | 103802408 | -0.019 | 0.003 | 293723 | 6.97E-13 | 0.3489 | G | A | Years of schooling | ieu-a-1001 |
| rs11588857 | 1 | 204587047 | 0.022 | 0.003 | 293723 | 1.31E-12 | 0.209 | A | G | Years of schooling | ieu-a-1001 |
| rs4800490 | 18 | 21126081 | 0.015 | 0.002 | 293723 | 2.13E-09 | 0.4571 | C | A | Years of schooling | ieu-a-1001 |
| rs28420834 | 15 | 82513121 | 0.015 | 0.003 | 293723 | 1.55E-09 | 0.5709 | G | A | Years of schooling | ieu-a-1001 |
| rs17824247 | 2 | 144152539 | 0.018 | 0.003 | 293723 | 5.29E-13 | 0.4198 | C | T | Years of schooling | ieu-a-1001 |
| rs7964899 | 12 | 14595756 | 0.017 | 0.002 | 293723 | 1.99E-11 | 0.4571 | A | G | Years of schooling | ieu-a-1001 |
| rs1035578 | 16 | 12531365 | -0.013 | 0.002 | 293723 | 4.71E-08 | 0.569 | A | G | Years of schooling | ieu-a-1001 |
| rs523934 | 11 | 95641775 | 0.015 | 0.003 | 293723 | 1.09E-08 | 0.416 | A | G | Years of schooling | ieu-a-1001 |
| rs1396967 | 10 | 64839820 | 0.015 | 0.003 | 293723 | 2.90E-09 | 0.3937 | C | T | Years of schooling | ieu-a-1001 |
| rs34305371 | 1 | 72733610 | 0.036 | 0.004 | 293723 | 2.34E-16 | 0.08769 | A | G | Years of schooling | ieu-a-1001 |
| rs7757476 | 6 | 14711961 | 0.02 | 0.003 | 293723 | 9.37E-10 | 0.2313 | A | G | Years of schooling | ieu-a-1001 |
| rs61160187 | 5 | 60111579 | 0.018 | 0.003 | 293723 | 5.93E-13 | 0.3806 | G | A | Years of schooling | ieu-a-1001 |
| rs538628 | 17 | 44787313 | -0.018 | 0.003 | 293723 | 2.16E-08 | 0.2444 | C | G | Years of schooling | ieu-a-1001 |
| rs9739070 | 12 | 123771032 | -0.024 | 0.003 | 293723 | 3.95E-16 | 0.7705 | G | A | Years of schooling | ieu-a-1001 |
| rs9792504 | 9 | 1751550 | 0.018 | 0.003 | 293723 | 3.25E-12 | 0.306 | G | A | Years of schooling | ieu-a-1001 |
| rs4240470 | 9 | 124621729 | 0.016 | 0.003 | 293723 | 2.63E-09 | 0.7071 | C | G | Years of schooling | ieu-a-1001 |
| rs320700 | 7 | 137049477 | 0.016 | 0.003 | 293723 | 1.50E-09 | 0.6343 | A | G | Years of schooling | ieu-a-1001 |
| rs12514965 | 5 | 113988893 | -0.018 | 0.003 | 293723 | 5.12E-10 | 0.2612 | C | T | Years of schooling | ieu-a-1001 |
| rs58694847 | 14 | 84916511 | -0.018 | 0.003 | 293723 | 7.41E-11 | 0.3097 | C | G | Years of schooling | ieu-a-1001 |
| rs4468571 | 15 | 78020132 | 0.014 | 0.003 | 293723 | 2.59E-08 | 0.4235 | G | A | Years of schooling | ieu-a-1001 |
| rs13010288 | 2 | 51824512 | 0.02 | 0.004 | 293723 | 2.21E-08 | 0.1119 | T | G | Years of schooling | ieu-a-1001 |
| rs13421974 | 2 | 155488869 | -0.014 | 0.002 | 293723 | 8.96E-09 | 0.4776 | C | T | Years of schooling | ieu-a-1001 |
| rs62263923 | 3 | 85674790 | 0.016 | 0.003 | 293723 | 1.63E-09 | 0.3563 | G | A | Years of schooling | ieu-a-1001 |
| rs16845580 | 2 | 161920884 | -0.016 | 0.003 | 293723 | 2.07E-10 | 0.3694 | C | T | Years of schooling | ieu-a-1001 |
| rs12410444 | 1 | 44188719 | 0.018 | 0.003 | 293723 | 2.14E-11 | 0.2817 | G | A | Years of schooling | ieu-a-1001 |
| rs6882046 | 5 | 87968864 | 0.021 | 0.003 | 293723 | 7.92E-14 | 0.3134 | G | A | Years of schooling | ieu-a-1001 |
| rs6839705 | 4 | 106144735 | -0.017 | 0.003 | 293723 | 1.72E-11 | 0.6399 | C | A | Years of schooling | ieu-a-1001 |
| rs766406 | 6 | 26319588 | 0.014 | 0.003 | 293723 | 1.89E-08 | 0.6194 | T | G | Years of schooling | ieu-a-1001 |
| rs12900061 | 15 | 66009248 | 0.021 | 0.003 | 293723 | 2.46E-10 | 0.1623 | A | G | Years of schooling | ieu-a-1001 |
| rs9527702 | 13 | 58384392 | -0.023 | 0.003 | 293723 | 4.99E-17 | 0.2369 | G | A | Years of schooling | ieu-a-1001 |
| rs4741351 | 9 | 14222782 | 0.017 | 0.003 | 293723 | 1.13E-09 | 0.6903 | G | A | Years of schooling | ieu-a-1001 |
| rs17425572 | 9 | 88006338 | -0.014 | 0.002 | 293723 | 4.58E-08 | 0.5597 | G | A | Years of schooling | ieu-a-1001 |
| rs11222416 | 11 | 130854650 | -0.015 | 0.003 | 293723 | 1.17E-08 | 0.416 | T | C | Years of schooling | ieu-a-1001 |
| rs12761761 | 10 | 133775375 | 0.017 | 0.003 | 293723 | 3.19E-08 | 0.2071 | T | C | Years of schooling | ieu-a-1001 |
| rs1008078 | 1 | 91189731 | -0.016 | 0.003 | 293723 | 7.88E-11 | 0.3731 | T | C | Years of schooling | ieu-a-1001 |
| rs11130222 | 3 | 49901060 | -0.026 | 0.003 | 293723 | 4.58E-25 | 0.4235 | T | A | Years of schooling | ieu-a-1001 |
| rs28792186 | 6 | 98508087 | 0.025 | 0.003 | 293723 | 6.40E-22 | 0.3955 | C | T | Years of schooling | ieu-a-1001 |
| rs71537331 | 1 | 243418182 | -0.017 | 0.003 | 293723 | 2.38E-10 | 0.3713 | T | C | Years of schooling | ieu-a-1001 |
| rs11726992 | 4 | 159668168 | -0.014 | 0.003 | 293723 | 2.58E-08 | 0.3545 | C | T | Years of schooling | ieu-a-1001 |
| rs35771425 | 1 | 211609768 | -0.019 | 0.003 | 293723 | 2.61E-10 | 0.2052 | C | T | Years of schooling | ieu-a-1001 |
| rs10006235 | 4 | 130670108 | 0.015 | 0.003 | 293723 | 2.63E-08 | 0.7127 | C | T | Years of schooling | ieu-a-1001 |
| rs34344888 | 14 | 23387585 | 0.016 | 0.003 | 293723 | 1.11E-10 | 0.6026 | G | A | Years of schooling | ieu-a-1001 |
| rs2456973 | 12 | 56416928 | 0.018 | 0.003 | 293723 | 1.58E-12 | 0.3209 | C | A | Years of schooling | ieu-a-1001 |
| rs7948975 | 11 | 90424638 | -0.014 | 0.003 | 293723 | 3.83E-08 | 0.3414 | C | T | Years of schooling | ieu-a-1001 |
| rs141979783 | 20 | 47788775 | 0.037 | 0.006 | 293723 | 6.67E-09 | 0.05224 | T | C | Years of schooling | ieu-a-1001 |
| rs9616906 | 22 | 51104680 | 0.015 | 0.003 | 293723 | 1.73E-09 | 0.4515 | A | G | Years of schooling | ieu-a-1001 |
| rs1382358 | 19 | 13171424 | -0.021 | 0.004 | 293723 | 3.17E-08 | 0.09328 | C | T | Years of schooling | ieu-a-1001 |
| rs12962421 | 18 | 44787498 | 0.014 | 0.002 | 293723 | 1.50E-08 | 0.4627 | G | A | Years of schooling | ieu-a-1001 |
| rs32940 | 5 | 141132286 | 0.0211431 | 0.0028965 | 397751 | 2.90E-13 | 0.700979 | C | T | Average total household income before tax | ukb-b-7408 |
| rs11588857 | 1 | 204587047 | 0.0213402 | 0.0032462 | 397751 | 4.90E-11 | 0.209469 | A | G | Average total household income before tax | ukb-b-7408 |
| rs12692596 | 2 | 161265910 | -0.0154093 | 0.0027335 | 397751 | 1.70E-08 | 0.3717 | T | C | Average total household income before tax | ukb-b-7408 |
| rs6699397 | 1 | 91212216 | -0.0190757 | 0.0027415 | 397751 | 3.50E-12 | 0.369644 | G | A | Average total household income before tax | ukb-b-7408 |
| rs10761035 | 9 | 99236092 | 0.0186402 | 0.0033851 | 397751 | 3.70E-08 | 0.188004 | A | G | Average total household income before tax | ukb-b-7408 |
| rs488786 | 1 | 20888207 | 0.0206878 | 0.0035676 | 397751 | 6.70E-09 | 0.164058 | T | C | Average total household income before tax | ukb-b-7408 |
| rs2068428 | 9 | 1792147 | 0.0169724 | 0.0030952 | 397751 | 4.20E-08 | 0.239041 | T | C | Average total household income before tax | ukb-b-7408 |
| rs9891103 | 17 | 44091886 | -0.0234517 | 0.0031482 | 397751 | 9.40E-14 | 0.229018 | T | C | Average total household income before tax | ukb-b-7408 |
| rs34473884 | 10 | 133761285 | 0.017225 | 0.003056 | 397751 | 1.70E-08 | 0.248977 | A | G | Average total household income before tax | ukb-b-7408 |
| rs6035877 | 20 | 21512532 | -0.0145138 | 0.0026504 | 397751 | 4.30E-08 | 0.469244 | C | A | Average total household income before tax | ukb-b-7408 |
| rs5754738 | 22 | 34280249 | -0.0162464 | 0.0029204 | 397751 | 2.70E-08 | 0.709551 | G | A | Average total household income before tax | ukb-b-7408 |
| rs75413320 | 19 | 13149854 | -0.0261129 | 0.0042683 | 397751 | 9.50E-10 | 0.108161 | C | T | Average total household income before tax | ukb-b-7408 |
| rs2332719 | 3 | 123712966 | -0.0183042 | 0.0029532 | 397751 | 5.70E-10 | 0.278917 | G | A | Average total household income before tax | ukb-b-7408 |
| rs7700107 | 4 | 17880416 | -0.0228296 | 0.0038299 | 397751 | 2.50E-09 | 0.138299 | C | A | Average total household income before tax | ukb-b-7408 |
| rs2515919 | 6 | 31564167 | -0.0159385 | 0.0027354 | 397751 | 5.70E-09 | 0.369978 | G | A | Average total household income before tax | ukb-b-7408 |
| rs3130264 | 6 | 33301229 | -0.015448 | 0.0026431 | 397751 | 5.10E-09 | 0.513869 | G | C | Average total household income before tax | ukb-b-7408 |
| rs73015322 | 6 | 163857445 | -0.0271853 | 0.0049537 | 397751 | 4.10E-08 | 0.076734 | T | G | Average total household income before tax | ukb-b-7408 |
| rs2820314 | 1 | 201872209 | -0.0165601 | 0.0027886 | 397751 | 2.90E-09 | 0.338157 | C | A | Average total household income before tax | ukb-b-7408 |
| rs11165472 | 1 | 96200401 | -0.0152441 | 0.0026424 | 397751 | 8.00E-09 | 0.486343 | T | A | Average total household income before tax | ukb-b-7408 |
| rs10429582 | 9 | 23346850 | 0.0270261 | 0.0026841 | 397751 | 7.60E-24 | 0.416388 | C | T | Average total household income before tax | ukb-b-7408 |
| rs11191116 | 10 | 103555611 | -0.0163993 | 0.0027764 | 397751 | 3.50E-09 | 0.351782 | T | C | Average total household income before tax | ukb-b-7408 |
| rs12883788 | 14 | 33303540 | -0.0188574 | 0.0026631 | 397751 | 1.40E-12 | 0.459864 | T | C | Average total household income before tax | ukb-b-7408 |
| rs784256 | 18 | 53398626 | -0.0253813 | 0.0033968 | 397751 | 7.90E-14 | 0.810934 | A | G | Average total household income before tax | ukb-b-7408 |
| rs12531825 | 7 | 8005174 | -0.0258177 | 0.0040617 | 397751 | 2.10E-10 | 0.122596 | A | G | Average total household income before tax | ukb-b-7408 |
| rs968050 | 6 | 98574560 | 0.0223829 | 0.0026507 | 397751 | 3.10E-17 | 0.483185 | T | C | Average total household income before tax | ukb-b-7408 |
| rs387780 | 2 | 32502495 | 0.0167299 | 0.0028273 | 397751 | 3.30E-09 | 0.674498 | C | T | Average total household income before tax | ukb-b-7408 |
| rs11678501 | 2 | 188919210 | -0.0517926 | 0.0085165 | 397751 | 1.20E-09 | 0.024727 | C | T | Average total household income before tax | ukb-b-7408 |
| rs2362523 | 19 | 5002301 | 0.0158667 | 0.0027984 | 397751 | 1.40E-08 | 0.339926 | G | A | Average total household income before tax | ukb-b-7408 |
| rs13002946 | 2 | 100801959 | 0.0205047 | 0.0029929 | 397751 | 7.30E-12 | 0.269216 | A | T | Average total household income before tax | ukb-b-7408 |
| rs62183028 | 2 | 212631483 | -0.0189832 | 0.0028611 | 397751 | 3.20E-11 | 0.310562 | T | G | Average total household income before tax | ukb-b-7408 |
| rs77126132 | 7 | 54966738 | 0.0267483 | 0.0045792 | 397751 | 5.20E-09 | 0.093289 | A | G | Average total household income before tax | ukb-b-7408 |
| rs11714337 | 3 | 71582521 | 0.0153807 | 0.0026788 | 397751 | 9.40E-09 | 0.431007 | A | G | Average total household income before tax | ukb-b-7408 |
| rs1455350 | 2 | 199497115 | -0.0172341 | 0.0026596 | 397751 | 9.20E-11 | 0.477454 | A | T | Average total household income before tax | ukb-b-7408 |
| rs6429636 | 1 | 44183540 | 0.0192858 | 0.0029352 | 397751 | 5.00E-11 | 0.719691 | T | G | Average total household income before tax | ukb-b-7408 |
| rs2422859 | 20 | 3132828 | 0.0163711 | 0.0026472 | 397751 | 6.20E-10 | 0.471922 | G | T | Average total household income before tax | ukb-b-7408 |
| rs7896518 | 10 | 65104500 | 0.014813 | 0.0027005 | 397751 | 4.10E-08 | 0.428029 | G | A | Average total household income before tax | ukb-b-7408 |
| rs1421334 | 8 | 30865733 | 0.0162948 | 0.0026703 | 397751 | 1.00E-09 | 0.549035 | C | A | Average total household income before tax | ukb-b-7408 |
| rs6868457 | 5 | 60550041 | 0.0209797 | 0.0026601 | 397751 | 3.10E-15 | 0.478238 | C | T | Average total household income before tax | ukb-b-7408 |
| rs71576284 | 7 | 126244536 | -0.0838859 | 0.0151769 | 397751 | 3.30E-08 | 0.008961 | A | C | Average total household income before tax | ukb-b-7408 |
| rs9388490 | 6 | 126704795 | 0.015105 | 0.0026686 | 397751 | 1.50E-08 | 0.439901 | T | C | Average total household income before tax | ukb-b-7408 |
| rs1229984 | 4 | 100239319 | -0.0491072 | 0.0080479 | 397751 | 1.00E-09 | 0.972949 | C | T | Average total household income before tax | ukb-b-7408 |
| rs4115668 | 16 | 28607532 | -0.0179965 | 0.0027917 | 397751 | 1.10E-10 | 0.346945 | A | G | Average total household income before tax | ukb-b-7408 |
| rs1079866 | 7 | 41470093 | 0.072 | 0.0084436 | 182416 | 1.50E-17 | 0.12 | G | C | Age at menarche | ieu-a-1095 |
| rs4242496 | 8 | 4822246 | -0.033 | 0.0056962 | 182416 | 6.90E-09 | 0.5 | A | T | Age at menarche | ieu-a-1095 |
| rs10840031 | 11 | 8410621 | 0.038 | 0.0064064 | 182416 | 3.00E-09 | 0.24 | A | G | Age at menarche | ieu-a-1095 |
| rs6758290 | 2 | 105864826 | -0.04 | 0.0063221 | 182416 | 2.50E-10 | 0.51 | C | T | Age at menarche | ieu-a-1095 |
| rs3733632 | 4 | 104640935 | 0.049 | 0.0078624 | 182416 | 4.60E-10 | 0.23 | G | A | Age at menarche | ieu-a-1095 |
| rs7642134 | 3 | 86916882 | 0.038 | 0.0059124 | 182416 | 1.30E-10 | 0.56 | G | A | Age at menarche | ieu-a-1095 |
| rs17351680 | 3 | 132586002 | 0.044 | 0.007557 | 182416 | 5.80E-09 | 0.13 | G | C | Age at menarche | ieu-a-1095 |
| rs633715 | 1 | 177852580 | -0.051 | 0.00725 | 182416 | 2.00E-12 | 0.2 | C | T | Age at menarche | ieu-a-1095 |
| rs10144321 | 14 | 100882405 | -0.042 | 0.0066176 | 182416 | 2.20E-10 | 0.22 | G | A | Age at menarche | ieu-a-1095 |
| rs3743266 | 15 | 60781513 | -0.045 | 0.0062327 | 182416 | 5.20E-13 | 0.33 | C | T | Age at menarche | ieu-a-1095 |
| rs913588 | 9 | 7174673 | -0.034 | 0.0056834 | 182416 | 2.20E-09 | 0.55 | A | G | Age at menarche | ieu-a-1095 |
| rs1874984 | 10 | 1731871 | 0.037 | 0.0060049 | 182416 | 7.20E-10 | 0.44 | C | G | Age at menarche | ieu-a-1095 |
| rs12003641 | 9 | 114279497 | 0.082 | 0.0105545 | 182416 | 7.90E-15 | 0.07 | T | C | Age at menarche | ieu-a-1095 |
| rs16938437 | 11 | 46052575 | -0.067 | 0.010348 | 182416 | 9.50E-11 | 0.05 | T | C | Age at menarche | ieu-a-1095 |
| rs1518080 | 2 | 199635382 | -0.051 | 0.0060044 | 182416 | 2.00E-17 | 0.43 | G | C | Age at menarche | ieu-a-1095 |
| rs6694738 | 1 | 243715210 | -0.044 | 0.0078848 | 182416 | 2.40E-08 | 0.18 | A | C | Age at menarche | ieu-a-1095 |
| rs1659127 | 16 | 14388305 | 0.044 | 0.0063717 | 182416 | 5.00E-12 | 0.3 | A | G | Age at menarche | ieu-a-1095 |
| rs852069 | 20 | 17122593 | 0.036 | 0.0059073 | 182416 | 1.10E-09 | 0.62 | G | A | Age at menarche | ieu-a-1095 |
| rs3115627 | 6 | 29820278 | 0.038 | 0.0064534 | 182416 | 3.90E-09 | 0.4 | G | A | Age at menarche | ieu-a-1095 |
| rs4840086 | 6 | 100208438 | -0.036 | 0.0056203 | 182416 | 1.50E-10 | 0.48 | G | A | Age at menarche | ieu-a-1095 |
| rs11022756 | 11 | 13315439 | -0.048 | 0.0064811 | 182416 | 1.30E-13 | 0.74 | C | A | Age at menarche | ieu-a-1095 |
| rs9997604 | 4 | 28752932 | 0.039 | 0.006511 | 182416 | 2.10E-09 | 0.7 | C | A | Age at menarche | ieu-a-1095 |
| rs466639 | 1 | 165394882 | 0.075 | 0.0087058 | 182416 | 7.00E-18 | 0.87 | C | T | Age at menarche | ieu-a-1095 |
| rs4369815 | 2 | 157126964 | -0.08 | 0.0118876 | 182416 | 1.70E-11 | 0.05 | G | T | Age at menarche | ieu-a-1095 |
| rs10483727 | 14 | 61072875 | -0.037 | 0.0057853 | 182416 | 1.60E-10 | 0.64 | C | T | Age at menarche | ieu-a-1095 |
| rs12148769 | 15 | 24152094 | -0.055 | 0.0097517 | 182416 | 1.70E-08 | 0.12 | A | G | Age at menarche | ieu-a-1095 |
| rs9635759 | 17 | 49613785 | 0.058 | 0.0063615 | 182416 | 7.70E-20 | 0.33 | A | G | Age at menarche | ieu-a-1095 |
| rs7103411 | 11 | 27700125 | -0.043 | 0.0070559 | 182416 | 1.10E-09 | 0.78 | T | C | Age at menarche | ieu-a-1095 |
| rs2836950 | 21 | 40604429 | -0.035 | 0.0062056 | 182416 | 1.70E-08 | 0.32 | G | C | Age at menarche | ieu-a-1095 |
| rs1516883 | 9 | 108907267 | -0.091 | 0.0024551 | 182416 | 1.00E-200 | 0.3 | A | G | Age at menarche | ieu-a-1095 |
| rs12291726 | 11 | 78088210 | 0.057 | 0.0083329 | 182416 | 7.90E-12 | 0.11 | G | A | Age at menarche | ieu-a-1095 |
| rs7853970 | 9 | 86715566 | -0.037 | 0.0062595 | 182416 | 3.40E-09 | 0.57 | C | T | Age at menarche | ieu-a-1095 |
| rs10938397 | 4 | 45182527 | -0.038 | 0.0059228 | 182416 | 1.40E-10 | 0.43 | G | A | Age at menarche | ieu-a-1095 |
| rs11767400 | 7 | 122160742 | 0.035 | 0.0062951 | 182416 | 2.70E-08 | 0.31 | A | C | Age at menarche | ieu-a-1095 |
| rs12598642 | 16 | 69848772 | 0.044 | 0.0056805 | 182416 | 9.50E-15 | 0.44 | G | A | Age at menarche | ieu-a-1095 |
| rs888345 | 8 | 140646990 | -0.044 | 0.0073152 | 182416 | 1.80E-09 | 0.82 | A | G | Age at menarche | ieu-a-1095 |
| rs13179411 | 5 | 133900513 | 0.06 | 0.0078132 | 182416 | 1.60E-14 | 0.16 | T | G | Age at menarche | ieu-a-1095 |
| rs9647570 | 5 | 167370263 | 0.046 | 0.0084271 | 182416 | 4.80E-08 | 0.12 | G | T | Age at menarche | ieu-a-1095 |
| rs2184968 | 6 | 126760994 | -0.036 | 0.0056723 | 182416 | 2.20E-10 | 0.41 | C | T | Age at menarche | ieu-a-1095 |
| rs740077 | 5 | 137760083 | -0.046 | 0.007045 | 182416 | 6.60E-11 | 0.21 | C | A | Age at menarche | ieu-a-1095 |
| rs13215865 | 6 | 56753730 | -0.042 | 0.0074722 | 182416 | 1.90E-08 | 0.15 | T | C | Age at menarche | ieu-a-1095 |
| rs7119712 | 11 | 101427771 | -0.041 | 0.0064452 | 182416 | 2.00E-10 | 0.19 | A | G | Age at menarche | ieu-a-1095 |
| rs2687729 | 3 | 127895226 | 0.044 | 0.0065749 | 182416 | 2.20E-11 | 0.23 | G | A | Age at menarche | ieu-a-1095 |
| rs2947411 | 2 | 614168 | -0.052 | 0.0076552 | 182416 | 1.10E-11 | 0.87 | G | A | Age at menarche | ieu-a-1095 |
| rs895526 | 2 | 200162425 | 0.044 | 0.0075532 | 182416 | 5.70E-09 | 0.8 | C | T | Age at menarche | ieu-a-1095 |
| rs618678 | 1 | 44133299 | -0.034 | 0.0061481 | 182416 | 3.20E-08 | 0.33 | T | C | Age at menarche | ieu-a-1095 |
| rs2344508 | 1 | 74999713 | 0.034 | 0.0057472 | 182416 | 3.30E-09 | 0.56 | A | G | Age at menarche | ieu-a-1095 |
| rs3914188 | 3 | 184010048 | 0.044 | 0.0067021 | 182416 | 5.20E-11 | 0.74 | C | G | Age at menarche | ieu-a-1095 |
| rs2153127 | 6 | 105348544 | -0.077 | 0.0020774 | 182416 | 1.00E-200 | 0.46 | C | T | Age at menarche | ieu-a-1095 |
| rs6933660 | 6 | 151803754 | -0.036 | 0.0063313 | 182416 | 1.30E-08 | 0.3 | A | C | Age at menarche | ieu-a-1095 |
| rs2617056 | 8 | 4563910 | -0.036 | 0.0059334 | 182416 | 1.30E-09 | 0.39 | T | A | Age at menarche | ieu-a-1095 |
| rs2179786 | 6 | 54753340 | -0.039 | 0.0057197 | 182416 | 9.20E-12 | 0.32 | T | G | Age at menarche | ieu-a-1095 |
| rs11756454 | 6 | 41798578 | 0.034 | 0.0057033 | 182416 | 2.50E-09 | 0.45 | A | T | Age at menarche | ieu-a-1095 |
| rs9939609 | 16 | 53820527 | -0.042 | 0.0057203 | 182416 | 2.10E-13 | 0.45 | A | T | Age at menarche | ieu-a-1095 |
| rs2303100 | 19 | 9968434 | 0.038 | 0.0056839 | 182416 | 2.30E-11 | 0.55 | T | C | Age at menarche | ieu-a-1095 |
| rs4801589 | 19 | 58953398 | 0.032 | 0.0056278 | 182416 | 1.30E-08 | 0.43 | G | C | Age at menarche | ieu-a-1095 |
| rs12915845 | 15 | 89042467 | -0.035 | 0.0057289 | 182416 | 1.00E-09 | 0.42 | T | C | Age at menarche | ieu-a-1095 |
| rs9555810 | 13 | 112181437 | 0.047 | 0.0064921 | 182416 | 4.50E-13 | 0.26 | G | C | Age at menarche | ieu-a-1095 |
| rs9565073 | 13 | 74643919 | 0.034 | 0.0058807 | 182416 | 7.40E-09 | 0.52 | C | T | Age at menarche | ieu-a-1095 |
| rs7944630 | 11 | 122838844 | 0.047 | 0.005728 | 182416 | 2.30E-16 | 0.57 | A | G | Age at menarche | ieu-a-1095 |
| rs1482853 | 3 | 156798473 | -0.038 | 0.0060425 | 182416 | 3.20E-10 | 0.39 | A | C | Age at menarche | ieu-a-1095 |
| rs6770162 | 3 | 24711013 | 0.036 | 0.0057331 | 182416 | 3.40E-10 | 0.44 | A | G | Age at menarche | ieu-a-1095 |
| rs6747380 | 2 | 56587749 | 0.065 | 0.0076578 | 182416 | 2.10E-17 | 0.17 | A | G | Age at menarche | ieu-a-1095 |

**Abbreviations:** EAF, effect allele frequency; se, standard error.
